# Additive Manufacturing of Multi‐Scale Porous Soft Tissue Implants That Encourage Vascularization and Tissue Ingrowth

**DOI:** 10.1002/adhm.202100229

**Published:** 2021-06-24

**Authors:** Fergal B. Coulter, Ruth E. Levey, Scott T. Robinson, Eimear B. Dolan, Stefano Deotti, Michael Monaghan, Peter Dockery, Brian S. Coulter, Liam P. Burke, Aoife J. Lowery, Rachel Beatty, Ryan Paetzold, James J. Prendergast, Gabriella Bellavia, Stefania Straino, Francesca Cianfarani, Monica Salamone, Carmelo M. Bruno, Kevin M. Moerman, Giulio Ghersi, Garry P. Duffy, Eoin D. O'Cearbhaill

**Affiliations:** ^1^ UCD Centre for Biomedical Engineering School of Mechanical and Materials Engineering University College Dublin Dublin D04 V1W8 Ireland; ^2^ Discipline of Anatomy School of Medicine National University of Ireland Galway Galway H91 TK33 Ireland; ^3^ Advanced Materials and BioEngineering Research Centre (AMBER) Trinity College Dublin Dublin D02 E161 Ireland; ^4^ Biomedical Engineering College of Science and Engineering National University of Ireland Galway Galway H91 TK33 Ireland; ^5^ Department of Mechanical and Manufacturing Engineering Trinity College Dublin The University of Dublin Dublin D02 PN40 Ireland; ^6^ Soils and Analytical Services Department Teagasc, Johnstown Castle Research Centre Wexford Y35 FN73 Ireland; ^7^ Discipline of Bacteriology School of Medicine National University of Ireland Galway Galway H91 TK33 Ireland; ^8^ Discipline of Surgery The Lambe Institute National University of Ireland Galway Galway H91 TK33 Ireland; ^9^ Explora Biotech Srl G. Peroni 386 Rome 00131 Italy; ^10^ ABIEL srl viale delle Scienze ed.16 Palermo 90128 Italy; ^11^ Media Lab Massachusetts Institute of Technology Cambridge Massachusetts MA 02139‐4307 USA; ^12^ Dipartimento di Scienze e Tecnologie Biologiche Chimiche e Farmaceutiche Università degli Studi di Palermo Palermo 90133 Italy; ^13^ Regenerative Medicine Institute School of Medicine College of Medicine Nursing and Health Sciences National University of Ireland Galway Galway H91 TK33 Ireland; ^14^ UCD Conway Institute University College Dublin Dublin D04 V1W8 Ireland

**Keywords:** additive manufacturing, device‐tissue interaction, medical device coatings, medical grade silicone, soft tissue implants

## Abstract

Medical devices, such as silicone‐based prostheses designed for soft tissue implantation, often induce a suboptimal foreign‐body response which results in a hardened avascular fibrotic capsule around the device, often leading to patient discomfort or implant failure. Here, it is proposed that additive manufacturing techniques can be used to deposit durable coatings with multiscale porosity on soft tissue implant surfaces to promote optimal tissue integration. Specifically, the “liquid rope coil effect”, is exploited via direct ink writing, to create a controlled macro open‐pore architecture, including over highly curved surfaces, while adapting atomizing spray deposition of a silicone ink to create a microporous texture. The potential to tailor the degree of tissue integration and vascularization using these fabrication techniques is demonstrated through subdermal and submuscular implantation studies in rodent and porcine models respectively, illustrating the implant coating's potential applications in both traditional soft tissue prosthetics and active drug‐eluting devices.

## Graphical abstract

Porous silicone coatings are additively manufactured onto the surface of soft‐tissue implants, aiming to address the notorious challenges of long‐term tissue integration and fibrosis. The “liquid rope coil effect” via direct ink writing is combined with atomizing spray deposition to create a multi‐scale “rope coiled” silicone coating architecture. In vivo studies show that this coating encourages highly vascularised tissue ingrowth.

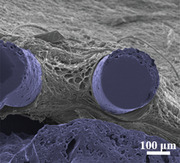

## 1 Introduction

Medical implants typically evoke a Foreign Body Response (FBR), which can be exacerbated around implants with a smooth surface.^[^ ^1^, ^2^ ^]^ FBR modulation is crucial for implanted devices requiring tissue integration and vascularization – for example, in cosmetic reconstruction,^[^ ^3^ ^]^ drug‐delivery,^[^ ^4^ ^]^ biosensors,^[^ ^5^ ^]^ and cellular‐encapsulation.^[^ ^6^ ^]^ Because of the FBR, medical implants are often enveloped by dense, avascular collagen layers, causing impaired tissue integration and vascularization. The FBR is problematic for cosmetic prostheses, resulting in constrictive capsular contraction and associated pain.^[^ ^7^ ^]^ At 10 years post‐implantation, capsular contraction occurs in 14.6% of breast augmentations, 28% of reconstructive surgeries, and 42% of revision surgeries.^[^ ^8^ ^]^

Promoting tissue integration and blood vessel formation is imperative for implant longevity and infection prevention.^[^ ^9^, ^10^, ^11^, ^12^ ^]^ Identifying materials and surface modifications that improve implant biocompatibility is an active research area.^[^ ^13^, ^14^, ^15^ ^]^ In the case of breast implants, 3D polyurethane open‐cell foam coatings were initially introduced as a means of encouraging tissue ingrowth and showed promise in significantly reducing rates of capsular contracture. However, concerns emerged over the long‐term stability of these polyurethane coatings and the release of a potential carcinogen, 2,4‐toluenediamine (2,4‐TDA), upon its degradation.^[^ ^8^, ^16^ ^]^ Interestingly, in a recent 30‐year follow‐up study on polyurethane‐coated breast implants, Castel et al. noted that capsular contracture rates (as measured by Baker II, III, and IV scores) remained at zero during the initial years of coating stability (estimated at 5.5 years post‐implantation), however, once the coating loses its macroscopic integrity, capsular contracture rates begin to climb from 8 years post‐surgery. Their conclusion was to recommend research to find a non‐toxic, non‐biodegradable synthetic material as an alternative to polyurethane.^[^ ^17^ ^]^

Textured silicone has been used to create breast implant coatings, with salt leaching techniques being adopted for surface macrotexturing. However, macro‐scale salt crystal leaching offers poor control of coating minimum wall thickness, increasing the risk of particulate shedding. Breast Implant Associated – Anaplastic Large‐Cell Lymphoma (BIA‐ALCL) is associated with macrotextured breast implants and may be a response to microparticulate debris.^[^ ^18^ ^]^ Such debris would be exacerbated by fabrication techniques that cannot control minimum wall thickness. Additive manufacturing controls the dimensions of deposited materials, resulting in coatings that are less prone to shedding.

Here, we demonstrate an additive manufacturing technique capable of rapidly depositing open‐porous silicone coatings featuring controllable porosity and curvature. Pores are created at both macroscale (>1 mm) and microscale (<20 µm). The former encourages tissue ingrowth without bridging fibrosis. The latter encourages cell attachment while reducing macrophage attachment and controlling fibroblast orientation.^[^ ^1^ ^]^

To generate the macro‐porosity, we employ the additive manufacturing technique “Direct Ink Writing” (DIW) and exploit the “Liquid Rope Coil” (LRC) effect – a phenomenon seen when a viscous fluid falls from a height onto a moving surface. Depending on the ratio of extrusion rate against speed of movement, the extrudate will create different looped geometry regimes.^[^ ^19^, ^20^, ^21^, ^22^, ^23^ ^]^ Substantial interconnected porosity is maintained along all major axes, which we hypothesize can improve tissue integration. We print open‐pore structures over highly curved surfaces, using multi‐axis printing techniques. Building upon pre‐existing principles,^[^ ^24^, ^25^ ^]^ we show that a substrate can be coated by first ascertaining the surface topology using laser measurements, and then calculating a toolpath over that surface – resulting in an evenly distributed coating. This shows that varying LRC loops can be used to completely coat complex shapes.

To generate a micro‐porous coating, we describe a formulation for a sprayable silicone ink, whereby a silicone elastomer is emulsified with a saturated saline solution using suitable surfactants. This low viscosity ink can be atomized to small droplets by use of a pressurized air carrier fluid, and sprayed as a coating with thickness in micro‐meter range. We examine the effects of depositing this micro‐coating both below and over the macro‐pore LRC coating. Spraying within an elevated temperature environment sees rapid solvent and then water evaporation, resulting in the nucleation of salt micro‐crystals which percolate throughout the silicone layer. As a final step, the silicone is cured and the crystals are removed through washing in water, leaving a microtopography over the surface of the coating. We show that by combining the two manufacturing techniques, it is possible to modulate tissue integration and significantly increase vascularization in a murine subcutaneous implantation model. Finally, we demonstrate the utility of the created devices as implantable prosthetics and drug‐delivery systems in a sub‐muscular porcine model.

## 2 Results

### 2.1 Macrotexture Control Using LRC

**Figure 1:**
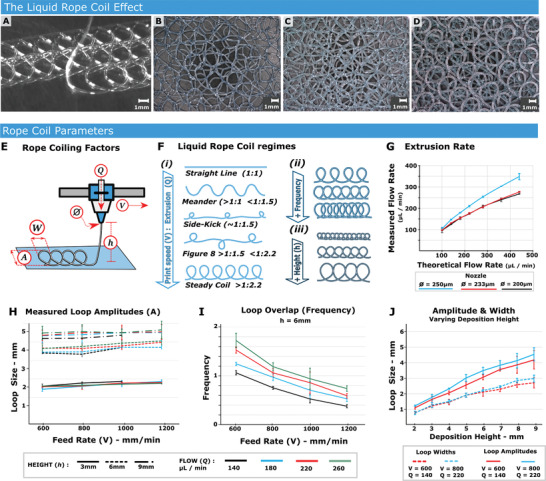
The liquid rope‐coil effect. A) Silicone loops deposited on a glass substrate. Scale bar = 1 mm. B) Three‐layer Liquid Rope Coil (LRC) coating, where the first layer is deposited along the printer *X* axis, second is placed at a 45°, and the third is at negative 45°. Scale bar = 1000 µm. C) Example of a six‐layer structure – essentially a twice replicated version of (B). Scale bar = 1000 µm. D) The underside of the six layer structure. Scale bar = 1000 µm. E) Schematic showing parameters which affect the shape, frequency and amplitude [*A*] of an extruded rope coil. [*Q*] is Material Flow, and [*Ø*] is nozzle diameter (the product of which can be used to calculate the extruded bead length per unit time), *V* is Print‐head velocity (Feed Rate). The nozzle height above substrate [*h*]. F) The ratio of [*V*] against extruded material filament length (per time) defines the loop regime/shape up to a certain threshold of 1:2.2, after which point a steady loop regime is reached and subsequent increase in material flow will affect the overlap or “loop frequency.” i) Illustrations of various loop regimes. ii) Example of increasing the loop frequency from 0.5 to 2. iii) Illustration of loops that would occur as the nozzle height [*h*] is increased. G) Extruded filament length plotted against the theoretical flow rate, *n* = 3. H) Measured loop amplitudes at various heights, Feed rates, and extrusion flow rates, *n* = 3. I) The loop overlap (analogous to frequency) for various extrusion rates and feed rates, when holding height steady in this case, [*h*] = 6, *n* = 3. J) Variation in loop amplitude and width when keeping feed and flow rates constant and varying only nozzle height [*h*], *n* = 3.

The LRC effect occurs when viscous material is extruded above a moving substrate. We show an example of depositing rows from a steady looped coil in **Figure** **1A** and Movie S1, Supporting Information. To create macroporous multilayer coatings, we extrude parallel rows of 3.5 mm amplitude loops to coat a substrate, followed by two additional layers created at 45° and then 135° to the first layer. A three‐layer coating is shown in Figure 1B and a six‐layer coating in Figure 1C.

To create LRC coatings, we first define print parameters specific to the material characteristics, using a 3D printer (Figure S1, Movie S1, Supporting Information). Material flow rate [*Q*], nozzle diameter [Ø], printhead/nozzle velocity [*V*], and nozzle height [*h*] all affect the buckling regime (Figure 1E). Numerous loop “regimes” can be created (Figure 1F (i)), the geometry of which is governed by the ratio between print‐head speed (or “Feed‐rate [*V*]) and the extrude rate [*Q*]. Here, we focus on the ‘Steady coil” regime (Figure 1F (i)), as this provides a repeatable and useful geometry for porous scaffolds and coatings with curved pore structure. When the “steady coil” regime is reached (ratio of ≈1:2.2, print‐head velocity: filament length), any flow rate increase will not change loop geometry, but increases loop overlap (termed “frequency”) (Figure 1F (ii)). Here we increase the frequency from 0.5 to 1 to 2. Increasing nozzle height with fixed flow rate increases the amplitude of extruded loops in a near‐linear fashion (Figure 1F (iii),J).

We verified printing constraints using biocompatible silicone (NuSil MED4820). This high viscosity material is capable of maintaining its extruded form – including unsupported bridging – without slumping. The extrusion was performed using a volumetrically controlled dispensing pump. The expected volume of extruded filament becomes non‐linear as extrude rates increase (Figure 1G). To confirm repeatability, numerous loops were deposited by varying flow rates, feed rates and heights, while measuring loop amplitude (Figure 1H). Amplitudes remained predictable as deposition height [*h*] and feed rate [*V*] varied. By holding the flow rate constant, and varying feed rate, the loop frequency can be varied linearly (Figure 1I), providing a simple method to decrease pore size during the print. We can also achieve linear increases in loop amplitude and width, by varying nozzle height (Figure 1J, Table S2, Supporting Information).

**Figure 2:**
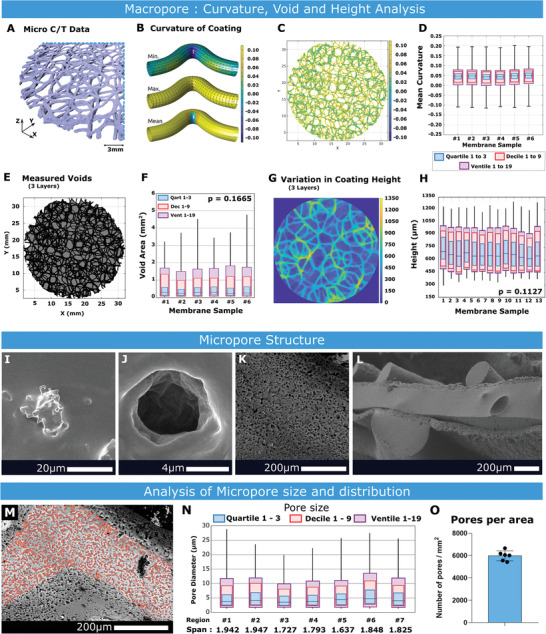
Macro and Micro pore analysis. A) Section of LRC coating segmented from a Micro C/T scan. Scale bar = 1 mm. B) A schematic visualization of minimum, maximum, and mean curvature for an example filament. C) LRC coating visualizing mean curvature. D) Mean curvature across six samples of LRC coating, each boxplot showing Quartiles, Deciles, and Ventiles, *n* = 6. E) LRC coating showing deposited material (black) and voids (grey). F) 2D negative voids in the coating, *n* = 6. G) Representative image of LRC Coating height map. H) Height variation of LRC coatings across samples, featuring plots of Quartiles, Deciles, and Ventiles, *n* = 13. I) Nucleated salt crystal following spray deposition. J) Pore generated after washing. K) Surface topology of a rope‐coil filament after micro‐texturing over‐spray. L) Sandwich structure of implantable drug eluting pouch. M) Example Region of Interest (ROI). N) Distribution of pore sizes (in µm) across 7 ROIs, showing 50%, 90% and 95% quantiles, along with the calculated span of pore sizes, *n* = 7. O) Mean value for number of micro‐pores per area, *n* = 6.

**Figure 3:**
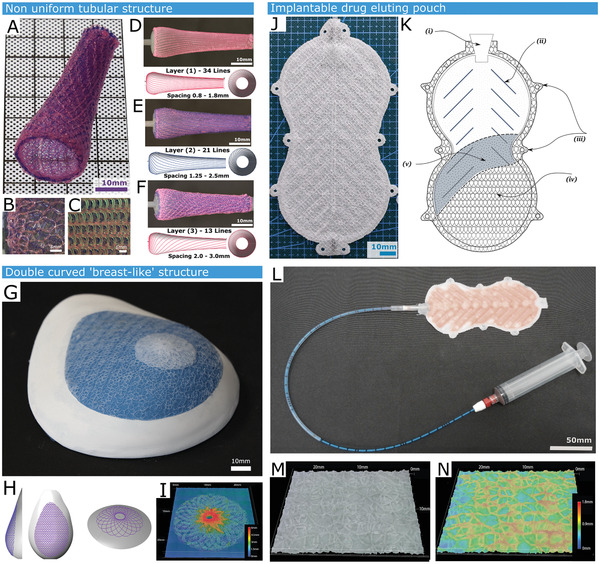
Demonstration of the versatility of liquid rope‐coiling in creating variable size porosity over different curved substrates. A) A non‐uniform circumference tubular structure demonstrating the ability to control deposition loop size. The three‐layer structure features a silicone rope coil. B) Close up of the structure from outside. C) Close up of structure from inside. D) Innermost layer featuring 34 helical lines with a spacing of 0.8 to 1.8mm. E) Middle layer featuring 21 lines of spacing 1.25–2.5mm. F) Outer layer featuring 13 lines spaced 2–3mm apart. G) A double curved surface form, replicating a silicone breast implant mold. This is 3D scanned to ascertain shape, spray coated with a 100 um layer of silicone, followed by a three layer of LRC Coating. H) Digital toolpaths corresponding to the LRC coating. I) The coiled layers can also feature shaping elements; in this case a height map shown a porous nipple and areola analog that was printed in the suitable location on the breast form. J) A Cellular Encapsulation device that features two adhered permeable microporous membranes fabricated by spraying our silicone/saline ink, then coating with LRC coating. K) Schematic of Encapsulation Device: (i) inner support structure (ii) surgical tie points (iii) outer rope‐coil layers (iv) input valve (v) permeable silicone membrane. L) Encapsulation device with attached filling catheter, filled with a hyaluronic acid / perfluorodecalin emulsion. M) Representative surface of the encapsulation device alongside N) the corresponding height map.

A repeatability assessment of the LRC coating technique was performed by analyzing LRC curvatures, 2D voids, and membrane height variation for coatings used on an exemplar implantable drug‐eluting pouch (Figures 3J–L, 8). Segmented voxel data for a section of three different Micro Computed Tomography (C/T) derived meshes were analyzed^[^ ^26^ ^]^ (**Figure** **2A**), allowing LRC surface geometry reconstruction and curvature and void analysis. A schematic for these curvatures illustrates the color scale corresponding to curvature degree (Figure 2B). Figure 2C presents a representative sample, and Figure 2D shows a boxplot of mean curvatures across samples. Curvature distribution was repeatable over measured samples.

Given that the three‐layer LRC coatings are relatively thin (<1.3 mm in height from substrate) relative to typical filament spacing (mean loop amplitude = 3.5 mm, width = 2.2 mm, filament = 0.2 mm), 3D void or porosity analysis could not be performed. 2D void analysis is presented instead. A representative image and boxplot showing measured voids across six samples are presented (Figure 2E,F). The mean void is 0.268 mm^2^ and while standard deviations are large, the confidence interval is repeatable across different samples.

Heights of the various extruded LRC filaments are measured in Figure 2G.

For coating mean curvature, 2D voids, and LRC height, we expected and observed variability; however, their mean and quantile distributions were similar between samples, demonstrating repeatability in the coating process (Figure S2 and Table S3, Supporting Information). Mechanical Testing of coating integrity under extension and shear was also examined, and this data can be seen in Figure S3, Supporting Information.

### 2.2 Microtexture Coating through Salt Nucleation

Surface textures in the 1–10 µm range are known to encourage cellular adhesion,^[^ ^1^, ^4^ ^]^ so to fabricate a microporous texture upon the substrate surface in this range, we developed an ink formulation which can be sprayed on an exposed surface through atomization. The ink is created by surfactant induced emulsification of a saturated saline solution with a medium shore hardness silicone (NuSil MED4840) in an organic solvent (*n*‐Heptane) solution. Specific details on spray ink formulation is given in Section 4.2.2.

When spraying the ink onto a substrate with an elevated temperature between 60 and 100 °C, there is near instantaneous evaporation of the heptane, followed by coagulation of the silicone. The trapped salt water droplets rapidly evaporate, leaving small salt crystals to nucleate throughout the uncured silicone. The salt to silicone ratio was increased to a point that while the crystals form, they join together, forming a percolating network. This means that the salt can be fully washed out. Empirically, it was found that a 2:1 ratio of the salt solution against the solid silicone content results in a reliably permeable and fully washable surface.

**Figure 4:**
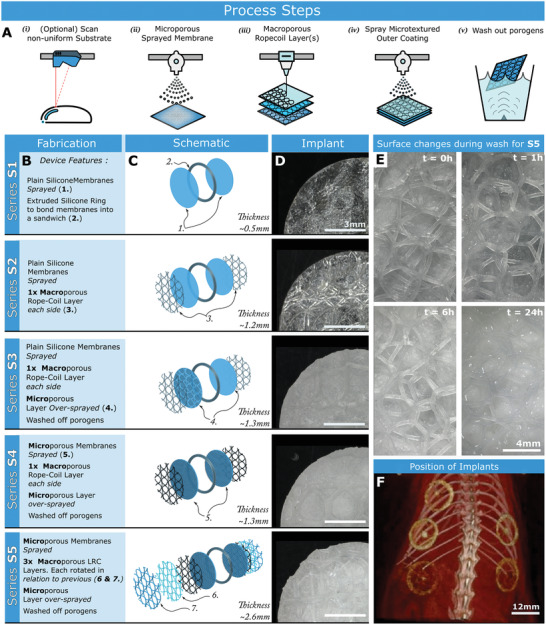
Combined manufacturing processes to test effects of increasing surface texture complexity. A) Fabrication steps required to create a conformal LRC Coating i) If substrate is a curved or a‐priori unknown shape, the geometry is scanned with measuring laser. ii) A Microporous membrane is sprayed on the substrate iii) LRC coating is extruded over the substrate surface. In this case 3 layers are deposited iv) A further microporous sprayed layer is added v) Fabricated device is sonicated in deionized water for 24 h. B) Fabrication steps for each type of tested implanted device (termed Series S1 to S5), each demonstrating an increased surface complexity. C) Schematic of each device. D) Implanted disk samples with a 12 mm diameter. E) Images showing the increasingly wetted surface of S5 over a period of ultrasonic washing. F) Positions of implants in the rat model.

Salt crystals still embedded in the silicone matrix after spraying and nucleation can be seen in Figure 2I, and a resultant pore is shown in Figure 2J. The sodium chloride crystals are removed over a 24‐h period washing in an ultrasonic bath with deionized water, after which the surface is heavily textured. Figure 2K shows an example micro‐textured surface. In Figure 2L a macro image of a two‐sided multi‐scale porous device is shown (corresponding with device S4 of Figure 4). A median pore size of 4.1 µm (st.dev 0.366 µm) over seven different samples was measured. A representative micropore region of interest is illustrated in Figure 2M, boxplot demonstrating distribution of pore size Figure 2N and graph of micropores per area in Figure 2O.

### 2.3 Macrotexture Deposition on Diverse Substrates

Three different technology demonstrators were produced. First, a non‐uniform tubular substrate was coated with progressively larger loops, illustrating versatile macropore size modulation while coating a generic design of a customizable endoprostheses (**Figure** **3A**‐F). The mandrel features a flared section.^[^ ^27^ ^]^ By calculating the distance between points on the toolpath and the neighboring line, the required nozzle offset [*h*] was calculated whereby printed loop rows would touch on upper and lower bounds. Three layers were printed in a helical fashion (Figure 3B‐F, Movie S1, Supporting Information). To maintain a constant velocity in the underlying substrate, a Constant Linear Velocity (CLV) approach was used.^[^ ^24^ ^]^

In a second application (coating a mammary implant), we coated the gypsum form of a human breast with a 250 µm thick silicone membrane (Figure 3G‐I). The substrate curvature was measured using a laser^[^ ^25^ ^]^ and a spray deposition toolpath was derived, ensuring that the nozzle tip is maintained at 85 mm from the surface. Three rope‐coil layers (*A* = 3.5 mm,) were then deposited over the surface (using NuSil MED 4820). On subsequent layers, parallel toolpath lines were rotated by ±45˚. A nipple structure and areolar complex were added to the substrate (Figure 3G) to demonstrate additive surface texturing that could be used when a nipple‐sparing mastectomy is not possible.^[^ ^28^ ^]^ Figure 3H,I shows printer toolpaths, a height map and a structure image.

The third demonstrator is an implantable drug eluting pouch, where a planar 100 µm porous silicone membrane is initially formed through layer‐by‐layer deposition of silicone/saline emulsion on a heated plate to create an interpenetrating salt crystal network that is subsequently solubilized and removed (Figure 3K (v),. A three layer LRC coating (4 mm loops) was deposited on the membrane (layer 1: NuSil MED4840, layer 2/3: 45° and 135° to the first (NuSil MED4820)) (Figure 3K (iv)). The entire membrane was oversprayed with the same ink, resulting in microtextured outer rope coils. Two such membranes were produced and cured (120 °C, 20 min). The membranes were positioned flat side up. An outline shape was extruded on one membrane using medium shore hardness silicone, and a one‐way input valve was affixed (Figure 3K (i–iii)). The second membrane was then overlaid, creating an internal cavity, and then cured (120 °C, 20 min). The cavity created in such a device could be filled with therapeutic agents before or after implantation, as demonstrated in Figures 3L and 8. A micrograph of the surface coating and corresponding height map is shown in Figures 3M and 3N respectively.

### 2.4 Macro/Micro Texturing Techniques for Varying Surface Complexity

Five different variations of disc‐shaped devices were fabricated for implantation and testing, each with an increasing level of micro and macro‐porosity. Starting with a pair of 12mm diameter plain silicone (NuSil MED4840) membranes sandwiched together (as a control series, termed S1), then adding an increasing level of macro and micro porous coatings for each subsequent series via LRC coating and spray respectively. **Figure** **4A**–C describe in full the differences in each series.

The morphological changes seen in the microtextured substrate of S5 after a 24 h period of washing in water is shown in Figure 4D, and the position of implants in the rodent model is shown in Figure 4F.

### 2.5 Surface Complexity Promotes Tissue Ingrowth

To assess the effect of macro/microporosity on the FBR in vivo, devices were implanted sub‐dermally in rats. Animals received one of each device (5 total), along with a sham surgical procedure (Figure 4F). After 2 weeks, the devices and surrounding tissue were analyzed.

**Figure 5:**
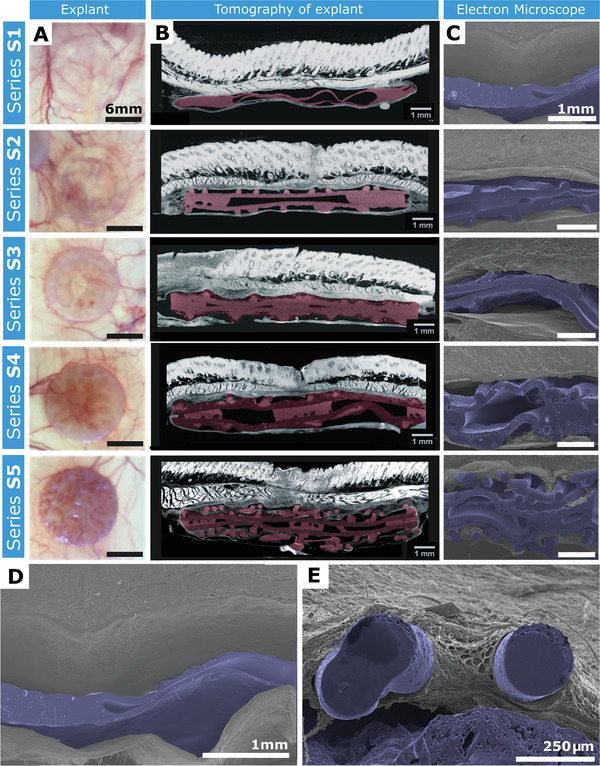
Post‐explantation representative images of devices in‐situ. A) Images of device S1–S5 post‐explantation on a light box allowing visualization of blood vessels and the tissue ingrowth surrounding the devices. Scale bar = 6 mm. B) Micro C/T cross sections of devices S1–S5 (colored pink). Scale bar = 1 mm. C) Scanning Electron Microscopy (SEM) cross‐sections of pseudo‐colored (blue) devices S1–S5. Scale bar = 1 mm. D) SEM image of device **S1** demonstrating no tissue integration. Scale bar = 1 mm. E) SEM image of device S5 demonstrating tissue integration surrounding rope coil. Scale bar = 250 µm.

Micro C/T analysis shows S1 and S2 appeared reasonably tissue‐free with little surface tissue incorporation (**Figure** **5A**). However, increasing microtexture in S3–S5 resulted in greater tissue incorporation (Figure S5, Supporting Information) with S5 demonstrating the greatest tissue incorporation (Figure 5B, Movie S2, Supporting Information). In S1 and S2, there were significant gaps between the device surface and surrounding tissue. The S3 surface displayed slightly better contour matching than S1 or S2, and both contour matching and tissue ingrowth increased further in S4. Tissue integration is less obvious in the electron microscope imagery of Figure 5C arising due to detachment following fixation of the tissue, but it is still visible that the multiple rope‐coiled pore layers result in excellent tissue ingrowth in S5 (Figure 5C). The device surface contours were imprinted in the tissue surrounding S3 and S4. The increase in tissue ingrowth is starkly obvious when comparing S1 and S5 (Figure 5D,E, Figure S5, Movie S2, Supporting Information).

### 2.6 Surface Texturing Controls Tissue Incorporation

**Figure 6:**
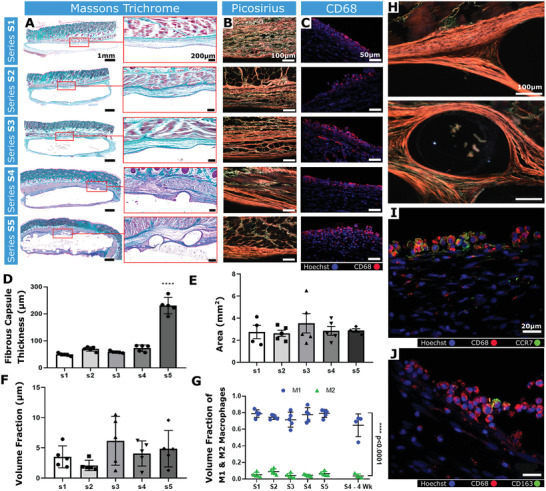
Analysis of the fibrous capsule composition and the immune response. A) Representative Masson's trichrome‐stained histological sections (Scale bar = 1 mm) and associated high magnification images of the fibrous capsule (Scale bars = 200 µm). B) Representative images of tissue‐device interfaces of S1–S5 imaged with polarized light microscopy after picrosirius red staining. Orange/red = mature collagen, Green = immature collagen. Scale bars = 100 µm. C) Representative immunofluorescent image of CD68+ cells. Scale bar = 20 µm. D) Mean fibrous capsule thickness. E) Areas of tissue extending from the tissue‐device interface to the panniculus carnosus. F) Volume fraction of CD68 + cells. G) Volume fraction of CCR7 and CD163 macrophages to total CD68+ at 2 weeks and S4 device at 4 weeks (*n* = 4). M1 versus M2 S1–S5 ****p < 0.0001. H) Polarized light microscopy images of rope‐coil feature on S4 and S5. Scale bars = 100 µm. I) Representative immunofluorescent image of CD68 and CCR7 (M1 phenotype marker). Scale bar = 20 µm. J) Representative immunofluorescent image of CD68 and CD163 (M2 phenotype marker). Scale bar = 20 µm. *n* = 4–5 per group; data are means ± SEM; ***p  < 0.001.

Differences in fibrous tissue were apparent between S1–S3 and S4, S5 (**Figure** **6A**). There was increased tissue ingrowth around the rope coil structures, indicative of newly developed tissue undergoing remodeling and this was strikingly apparent in S4 and S5 compared with S1–S3.

A significant increase in capsule thickness was observed with S5 (**** = *p *< 0.0001; Figure 6D). However, an area measurement of the fibrous tissue surrounding S1–S5 revealed that the area between the device interface and the superficial muscle layer remained unchanged (Figure 6E). This suggests that although capsule thickness significantly increased in S5, the overall amount of capsule was not increased, but appeared thicker due to a more tortuous development.

Isotropic collagen deposition was more evident in S1, S2 compared with S3–S5, which exhibited increasingly organized collagen (Figure 6B,H). The collagen was arranged into bundles orientated parallel to the device surface, forming a capsule of concentric layers. Most of the capsule in S4 and S5 stained red/orange (picrosirius red staining), implying the presence of mature collagen type I. However, in S1–S3 there was an increased proportion of green and yellow fibers (collagen type III‐like) indicative of remodelling.^[^ ^29^, ^30^, ^31^ ^]^ These data suggest that increasing the surface macrotexture promotes tissue ingrowth and implant integration in vivo.

### 2.7 Surface Texturing Does Not Increase Macrophage Abundance

Tissue sections stained with a pan‐macrophage marker (CD68) revealed no significant inter‐group differences (Figure 6C,F)). There was a higher ratio of CCR7+ macrophages (M1) compared with CD163+ macrophages (M2) across all groups, suggesting that most macrophages were pro‐inflammatory (Figure 6G,I,J)). A second cohort of S4 devices remained implanted for 4 weeks (*n* = 4), and demonstrated no difference in the CCR7+:CD163+ macrophage ratio (Figure 6G). These data indicate that increasingly complex topographies do not evoke enhanced macrophage responses.

### 2.8 Increased Surface Texturing Complexity Increases Blood Vessel Density

**Figure 7:**
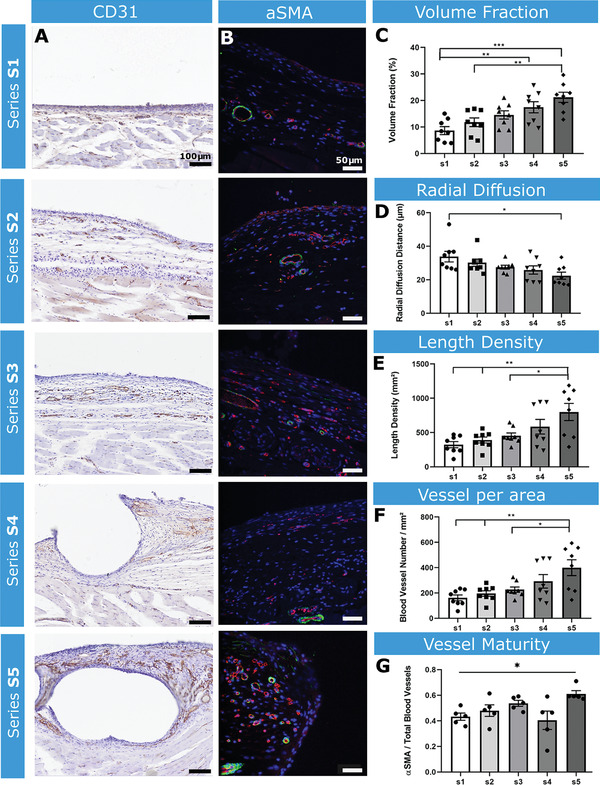
Analysis of angiogenesis and vessel maturity surrounding the implanted devices. A) Representative images of CD31 staining samples S1–S5. Scale bar = 100 µm. B) Representative fluorescent images of *α*‐SMA (green) and CD31 (red) staining of S1–S5 at the tissue‐device interface. Scale bar = 50 µm C) Volume fraction of blood vessels. D) Radial diffusion distance. E) Length density of blood vessels F) Number of vessels per area. G) Ratio of *α*‐SMA to CD31+ vessels. *n* = 5 per group; data are means ± SEM; *p < 0.05, **p < 0.01, ***p < 0.001.

Increasing surface architecture complexity led to significantly increased volume fraction, number per unit area, length density, and radial diffusion distances of blood vessels at the tissue‐device interface, between S1 and S5 (**Figure** **7A**,C–F).

The ratio of *α*‐Smooth Muscle Actin (*α*‐SMA: marker of vessel maturity) positive vessels was quantified, revealing a significant increase in *α*‐SMA+ vessels in S5 compared with S1 (Figure 7B,G), indicating that the complex surface of S5 led to blood vessel recruitment and maturation.

### 2.9 Scaling Surface Texturing for Large Animal Implants

We aimed to confirm that surface texturing augments tissue integration in a large animal model broadly mimicking a human breast implant. We also aimed to exploit microtexturing‐generated porosity in a drug‐delivery system. For a singular muscular implant (SMI), we desired a well vascularized fibrous capsule that permits drug diffusion. The S5 surface texture was selected, as this promoted increased CD31+ cells. The device was filled with a radiopaque hyaluronic acid (HA) emulsion as a drug‐delivery surrogate (Figure 3L).

### 2.10 SMI Functionality in Porcine Diabetes Model

**Figure 8:**
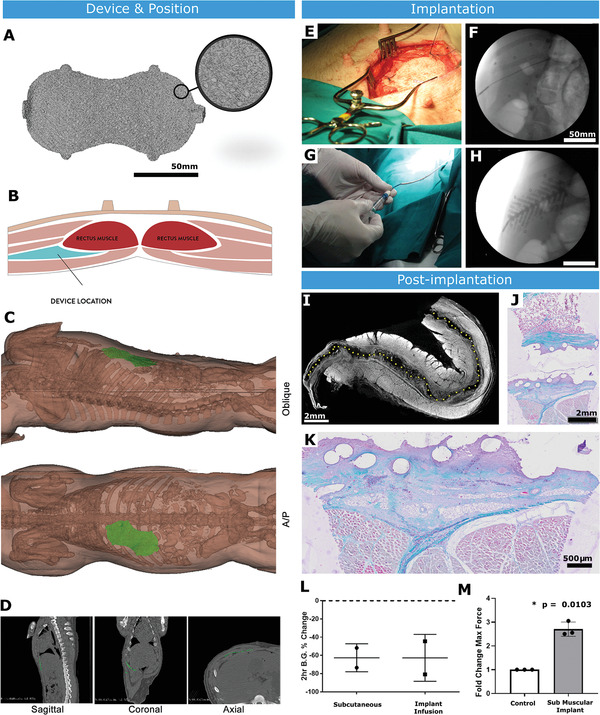
Submuscular implantation in a large animal model. A) A volumetric rendering of implant (previously shown in Figure 2) after Micro C/T imaging. B) Schematic of submuscular implant site in the anterior abdominal wall of pig. C) Schematic showing positioning of implant after surgery. D) C/T scans used to examine dimensions of AAW implantation site. E) Surgical placement of implant in submuscular plane. F) X‐ray fluoroscopy of device after implantation showing radiopaque markers to confirm positioning. Scale bar = 50 mm. G) Infusion of inner channel of implant with filling catheter. H) X‐ray fluoroscopy of implant filling with radiopaque hydrogel. Scale bar = 50 mm. I) Micro C/T of explanted device stained ex vivo with iodine (dotted line shows device space, m = muscle, fc = fibrous capsule) Scale bar = 2 mm. J) Trichrome stain of core biopsy taken through implant and surrounding tissue. Scale bar = 2 mm. K) Magnified image of trichrome stain demonstrating device integration into the surrounding tissue. Scale bar = 500 µm. L) Response of diabetic animals to insulin delivered through subcutaneous injection versus infusion through the implant. M) Pull‐off testing of control smooth silicone and textured implant after 2 weeks of submuscular implantation in pig. *n* = 2–3 per group; data are means ± SD; *p < 0.05.

The SMI was implanted between abdominal muscle layers (**Figure** **8B**; Figure S6, Supporting Information). Implantation and HA distribution were confirmed through fluoroscopic imaging (Figure 8F,H).

To assess tissue integration, streptozotocin (STZ)‐induced diabetic pigs received bilateral submuscular implants (*n* = 4). The SMIs were explanted after 2 weeks with surrounding muscle. Tissue ingrowth was noted, with excellent contour matching to the device surface (Figure 8I–K). The blood glucose change after 2 hours in animals that received SMI‐mediated insulin infusion was comparable to that achieved through gold standard subcutaneous delivery (Figure 8L).

In a pull‐off test, there was a significant fold change in the maximum tissue adhesion force between the SMI and surrounding tissue in the textured group compared with smooth silicone (*p = 0.0103; Figure 8M), indicating that S5 provides excellent tissue integration.

## 3 Discussion

Herein, we describe a method to create open porous structured coatings, at multiple length scales and featuring controlled minimum wall‐thickness. The coatings can be deposited onto non‐intersecting manifold surfaces through a combination of additive extrusion and spraying. Parameters required to create macropores (0.8–5 mm) were explored, and confirmed as highly repeatable. We demonstrated that macropore sizes can be varied mid‐print, resulting in open‐cell structures with variable density. Our direct deposition techniques display advantages over reported techniques for cellular scaffold fabrication,^[^ ^32^ ^]^ where precise control of minimum wall thickness is important. The rope‐coil deposition is rapid, and complex surfaces can be easily coated after their shape is ascertained. Furthermore, surface anomalies are accommodated, as the loops conform to diverse surface features.

Using spray deposition, we created micropores of <1–20 µm. Solvent casting and particulate leaching are limited in creating coating thickness (0.5–2 mm),^[^ ^33^ ^]^ facilitate minimal inter‐pore connectivity and surface texturing, and control porogen size rather than feature minimum wall‐thickness. These techniques are disadvantageous for pre‐implantation sterilisation and post‐implantation tissue ingrowth. Conversely, with our approach, microtexturing is limited to surfaces in the spray nozzle line‐of‐sight (Figure 2L). However, benefits of this microtexturing include enhanced wettability and tissue ingrowth (Figures 4E, 5B).

Macrotextured coatings have seen extensive use in breast reconstruction to reduce capsular contraction. While these surfacing techniques can improve implant stabilisation,^[^ ^34^ ^]^ poor control over minimum wall thickness can lead to particulate shedding.^[^ ^35^ ^]^ Randomly oriented pores lead to heterogeneous fibroblast orientation, creating multi‐vector forces that neutralise each other, resulting in softer implant capsules and reduced contraction.^[^ ^36^ ^]^ With our device, both the microtexture pore size and macrotextured rope coil surface could potentiate random fibroblast orientation, reducing contraction.

The improved structural integrity of the LRC technique should reduce microparticulate generation. Importantly, S5 endotoxin levels were undetectable, indicating effective sterilization.^[^ ^35^, ^36^, ^37^ ^]^

The geometry of the pores themselves are seen as an important factor for encouraging tissue in‐growth. We focus on looped filament regimes to create our overlapping LRC coatings, as opposed to the simple straight line and orthogonal grid structures commonly found in 3D printed scaffolds. This is justified by the knowledge that tissue growth begins in areas of large local negative curvature, then only commences growing across flat faces or straight lines (with zero curvature) when the neighborhood becomes curved due to tissue growing outward from the corners. As such, it can be said that cells can sense the curvature of their substrate at length scales much greater than themselves.^[^ ^38^ ^]^ The highly curved surfaces imparted by the LRC coating (Figure 2B–D) may be a contributory factor to the vascularized tissue in‐growth observed and warrants further study.

The macropore structure generated herein promotes tissue ingrowth, and as demonstrated, more complex surface texturing (devices S4 and S5) significantly enhanced tissue attachment compared with smooth implants. Furthermore, the S4 and S5 coatings promoted vascularization and enhanced vessel maturity without inducing a major immune response. This manufacturing technique is highly suited for drug‐delivery. Improved tissue integration and peri‐implant vascularity can enhance drug uptake, and the interconnected porous structures allow for drug diffusion. We demonstrated this by designing a submuscular implant for insulin delivery, showing excellent implant integration and a reduction in blood glucose following insulin delivery.

The S2–S4 surfaces demonstrate increased tissue integration compared with S1. This demonstrates that adding a micro/macrotexture can increase device integration. However, with multiscale porosity, an optimal response is obtained. The S4 device encouraged tissue integration without inducing a macrophage‐initiated immune response. However, S4 did not have the same effect as S5 on vascularity. While the volume fraction in S4 was greater than S1, vessel number, length density, or radial diffusion distance did not differ. This implies increased vessel size around the implant, but not increased vessel number. S4 may not encourage the degree of angiogenesis seen in S5, but the thinner capsule may aid drug‐delivery. Previously, we have shown an implantable device with a surface akin to S4 can be used as a controllable sustained release device^[^ ^39^ ^]^ Additionally, capsular contraction is correlated with capsular neo‐angiogenesis.^[^ ^40^ ^]^ Therefore, S5 may not be ideal for breast implants due to its rich vascular supply, while S4 could modulate fibroblast orientation and angiogenesis, reducing FBR and capsular contraction.

Attempts in reducing or eliminating these complications by surface modification of medical grade silicones with biomimetic compounds, such as phosphorylcholine, collagen I, or spider silk have also been tested.^[^ ^41^, ^42^, ^43^ ^]^ Phosphorylcholine‐coated silicone implants exhibit a decreased inflammatory reaction, and subsequent reduction of periprosthetic fibrosis.^[^ ^41^ ^]^ Hauser et al. demonstrated in‐vitro that a stable collagen I surface coating on silicone implants could potentially enhance cell affinity and biocompatibility of the material.^[^ ^42^ ^]^ Zeplin et al. utilized recombinant spider silk protein coating which improved implant biocompatibility by masking the implant's surface after implantation.^[^ ^43^ ^]^ All aforementioned coatings serve as alternative therapeutic strategies, however they lack the single material approach that our strategy offers.

The techniques described here represent fast, effective methods of coating medical implants with curved topologies, which can modulate the immune response and FBR. These patterning techniques offer tunability of tissue integration, capsule formation and angiogenesis. This methodology is simple and inexpensive for biomaterial surface functionalization.

## 4 Experimental Section

### Analysis of Macro and Micro Porosity—Analysis of Individual Loop Geometries

A series of extruded LRC lines were printed at feed rates 600, 800, 100, and 1200 mm min^−1^ each at the extrude rates: 140, 180, 220, and 260 µL min^−1^ using NuSil MED 4820 silicone. For each parameter set, three lines were printed. This was repeated for deposition: 3, 6, and 9mm. The amplitude and width of a randomly selected loop from each line was measured using a Keyence VHX 6000 measurement microscope. The values were recorded and analyzed by one way ANOVA in Microsoft Excel – See Table S2, Supporting Information.

*Analysis of Macro and Micro Porosity—Analysis of Mean Curvatures*: A subset of six samples were subjected to Micro C/T imaging (µCT 100, SCANCO Medical AG, Brüttisellen, Switzerland, 45 kVp, and 200 µA with a 0.1 mm aluminum filter,). The Micro C/T DICOM image data was imported into Mimics (18.0.0.525, Materialize, Leuven, Belgium), a graphical user interface based image viewing and segmentation tool, and the LRC coating structure was segmented based on thresholding. The binary (0 = background, 1 = LRC structure) segmented image data could then be exported to DICOM files. These binary DICOM files were then imported into MATLAB (The MathWorks R2020a, Natick, MA, USA) for further analysis using the GIBBON toolbox (https://www.gibboncode.org/26).

For curvature analysis, triangulated surface models were constructed for each of the six samples capturing the 3D LRC coating geometry. The surface models were derived from levelset images (using GIBBON's levelset2isosurface function) which in turn were computed using a Euclidean distance transform of the binary segmentation data (based on MATLAB's bwdist function). For each point on the surface, principle curvature analysis was performed (using GIBBON's patchCurvature function) providing the maximum and minimum curvature directions and amplitudes. Next mean curvature was computed as the mean of the minimum and maximum curvatures amplitude. Figure 2B illustrates an example filament‐like model shaded towards computed minimum (top), maximum (middle), and mean (bottom) curvature. To study repeatability of curvature, quantile ranges were computed for mean curvature for each of the six samples.

*Analysis of Macro and Micro Porosity—Analysis of 2D Void Size*: As described in Section 2.1, the aspect ratio of filament against loop size and layer height prevented meaningful performance of 3D void analysis. Instead, 2D void analysis was performed. First using C/T derived voxel data, a maximum intensity projection was computed across all slices, thereby producing a single binary 2D image for each sample. Figure 2E shows a representative image of this maximum intensity projection. Voids were defined as clusters of non‐filament, that is, background, pixels within these 2D images, and their size was computed from their surface area. To study repeatability of the 2D macro voids, mean and quantile ranges were computed for void size distributions for each of the six samples.

*Analysis of Macro and Micro Porosity—Analysis of LRC Coating Height*: To study the repeatability of the height of the LRC coatings, a total of 13 sample discs were cut (12 mm in diameter) from a subset of three different LRC coatings (prepared as per Sample S5). Spatially varying height maps were obtained for each isolated disc using the “Depth Up” feature of the Keyence VHX 6000 measurement microscope. Figure 2G illustrates a representative image for such a measurement. The acquired height maps for all discs were imported into MATLAB for mean, maximum and minimum height analysis.

*Analysis of Macro and Micro Porosity—Analysis of Micropore Size Distribution*: Six scanning electron microscope (SEM) images at 200× magnification were taken across three samples. One image focused on an area of membrane which did not feature any rope coil macrostructure. For analysis, a region of interest (ROI) for each image was created via a mask in Adobe Photoshop (CC 2018). This mask focused only on an area of the image where the pores were normal to the camera, thus avoiding any parallax effects from pores on a slope. The image featuring no rope‐coil structure was divided in two, to closer match the ROI area of the others. These images and corresponding ROIs are shown in Figure S4, Supporting Information. The ROIs were imported into ImageJ Fiji v1.52P, and each were batch‐processed by adjusting the Window/Level (*W* = 88, *L* = 141). This improved the contrast between the pores and non‐textured substrate. The “Analyze Particles” function with settings: Size – 0.38 µm^2^‐Infinity and Circularity 0.10–1.00 was used. This circularity setting removed multiple pores at the edges of the ROI, which were merged together by the thresholding function. The areas of all the pores were exported and analyzed in Microsoft Excel.

### Device Fabrication—Creation of Macroporous Layers

Implant grade silicones: Soft (Shore A20: NuSil MED4820) and Medium (Shore A40: Nusil MED4840) were combined Part A and B per datasheet. To aid visualization (for photographs only), a silicone pigment (Smooth‐On SilPig) was added at 0.5 wt% in various colors. The two‐part silicone was mixed in a planetary mixer (Thinky ARE‐310) for 10 minutes at 1850RPM, placed in a fridge for 30 minutes, and centrifuged at 4000 RPM for 5 min to remove air bubbles. The material was then loaded into an Viscotec ECOpen EC300 extruder mounted in a custom 3D printer (See Figure S1, Supporting Information). A 27 Gauge (200 µm I.D.) conical tip was used. Printing parameters are listed in supplementary materials.

*Device Fabrication—Sprayable Silicone Ink Material Preparation*: The salt water solution was formulated by heating DI water to 80 °C and adding 40 wt% Sodium Chloride salt (Sigma Aldrich) while stirring with a magnetic stirrer until fully saturated. The liquid was filtered through a 10 µm filter to remove any undissolved particles. A separate mixture of DI water with 20 wt% PEG 6000 was created by stirring at room temperature, and similarly filtered. The two mixtures were combined at a ratio of 3:1 in favor of the salt solution and this mixture was then filtered through a 0.22 µm syringe filter to remove contaminants. NuSil MED4840 Silicone was combined as Part A and B and mixed at a standard 1:1 ratio. This was then diluted using 99.1% n‐Heptane (Sigma Aldridge chemicals), at a ratio of 1:3 wt%. mixed in a planetary mixer for 10 min at 1850 rpm. For certain applications, blue pigment was added (0.1% Smooth‐On Ignite pigment) to aid visualization.

A surfactant blend with a hydrophilic/lipophilic balance (HLB) number of 11.5 was created by combining Span 85 (Sorbitan Trioleate) with Tween 40 (PEG‐20 Sorbitan Monopalmitate) at a ratio of 3:7. On combining the pore‐generating water solution with the silicone solution at a 1:3 ratios, the surfactant blend was added at a quantity of 3 wt% of overall liquid. The combination was mixed at 1750 rpm for 8 min in a planetary mixer. Nozzle settings for the spray solution is included in supplementary methods.

### Rodent Study

Rodent studies were approved by the Italian Minister of Health (Authorization No. 66/2017‐PR) and performed by Abiel Srl (Italy). Twelve‐week‐old, female RccHan Wistar rats (ENVIGO) received surgical implants. Rats were anesthetized by isofluorane and hair was removed. Five devices (S1–S5) were implanted via separate incision in the dorsal subcutaneous space and a 6th incision was included as a sham control. Implant sites were rotated on each animal to account for any site‐specific effects. Animals were treated with ceftriaxone (25 mg Kg^−1^) and tramadol (4 mg Kg^−1^), for 5 days. After 2 weeks, the animals were perfused with Iopamiro 370 (15 mL/hr) by cannulation (cannula 26G) at the level of a tail vein and analyzed by computerized axial tomography (Capiler CT‐Scannet, PerkinElmer).

### Fixation, Embedding, and Staining

For the rodent studies, animals were euthanized at 2 or 4 (S4 only) weeks, each device and the immediate surrounding tissue were extracted. Tissues were fixed in 4% paraformaldehyde (pH 7.4) then washed in 0.2 m phosphate‐buffered saline. Samples were transected, orientated and embedded in paraffin wax blocks. Sections of 5 µm were cut and stained with Masson's trichrome for fibrous capsule analysis. Additional sections were stained with a CD31 primary antibody (ab28364, Abcam, UK) and Mouse and Rabbit Specific HRP/DAB kit (ABC) Detection IHC kit (ab64264, Abcam, UK). Slides were imaged using an Olympus SlideScanner VS120 with an UPLSAPO 20× objective lens.

### Quantitative Analysis of the Fibrotic Capsule

A stereological method based on orthogonal intercepts was utilized^[^ ^44^ ^]^ to analyze the thickness of the fibrous capsule surrounding the implanted devices stained with Masson's Trichrome.^[^ ^45^ ^]^ From each tissue section, six non‐overlapping images were analyzed of the surrounding fibrous capsule at 4× magnification. Using Image J (Fiji version 2.0.0), a stereological square grid was superimposed onto the image stacks to provide test lines. Where the tissue‐device interface of the capsule intersected a test line, an orthogonal line was drawn from this point to the edge of the capsule and measured. These measurements were used to calculate the arithmetic mean thickness.

Area measurements were performed to estimate the area of the tissue spanning from the tissue device interface to the overlying panniculus carnosus muscle (used as a standard boundary line for each tissue section). Two tissue sections from each implant, from each animal were used. 2–3 non‐overlapping images at 1× magnification were required to gain full view of the surrounding fibrous capsule. Using Image J (Fiji version 2.0.0), the polygonal selection tool was used to delineate the boundaries of the area to be measured. Area was expressed in mm^2^.

### Polarized Light Microscopy

Paraffin embedded tissue sections were stained in 0.1% fast green (pH 7, Fast Green FCF; Sigma Aldrich) and 0.1% Sirius red in saturated picric acid (picrosirius red stain), according to previously established protocols.^[^ ^46^ ^]^ Polarized light micrographs were captured using an Olympus BX4 polarized light microscope (Mason Technology Ltd. Dublin, Ireland) at 20× magnification. Images were taken whereby maximum polarization was achieved by adjustment of the polarizing filters, and again orthogonal to this maximum polarization. The two captured images were merged using the MAX function in ImageJ software.

### Immunofluorescent Staining

Tissue sections were blocked with 10% goats’ serum / 1% BSA, then stained with a primary antibody followed by the appropriate secondary and a Hoechst nuclear stain was performed. Images were acquired with a spinning disc inverted confocal microscope (Yokagawa CSU22). 20 random fields of view of the capsule were collected, and images were processed using ImageJ. CCR7+ and CD163+ macrophages were counted and expressed as a ratio of total CD68+ macrophages. Blood vessels positive for *α*‐SMA in the capsule were counted with ImageJ and expressed as a ratio of total. An estimation of the volume fraction of myofibroblasts within 100 µm of the tissue device interface was carried out using a stereological grid. Intersections falling on *α*‐SMA+ cells were counted and expressed as a ratio of total intersections on the tissue, as per.^[^ ^47^ ^]^

### Statistical Analysis of Histomorphometrical Data

Data from fibrous capsule, angiogenesis analysis and vessel maturity analysis were tested for normality and equal variance. Data from both the fibrous capsule and angiogenesis studies were normally distributed, and of equal variance, were expressed as the mean ± SEM (Standard Error of the Mean). Multiple comparisons were made using analysis of variance (ANOVA). When differences were found, Tukey's post‐hoc test was used. Correlations were evaluated using GraphPad Prism 8.0.1. A *p* value of <0.05 was considered significant. Data from the vessel maturity analysis did not show equal variance, so a Kruskal‐Wallis test and Dunn's multiple comparisons tests was used.

### Micro C/T imaging of Subdermal Implants

Unstained explanted devices from rats were stored in 70% ethanol and imaged in a Micro C/T 100 scanner. Samples were scanned at 45 kVp and 200 µA with a 0.1 mm aluminum filter. Images and videos of each device iteration were generated with CTVox software (Bruker, USA). For soft tissue visualizations, samples were stained in a solution of 2.5% phosphomolybdic acid in 70% ethanol for 5–7days. Micro C/T images were captured using the Micro C/T 100 scanner at 70 kVp and 85 µA with a 0.5 mm aluminum filter. Images and videos were generated using ImageJ software.

### Scanning Electron Microscopy

Tissue samples were bisected longitudinally to create a cross‐section of the device and surrounding tissue. Samples were post‐fixed overnight in 2.5% glutaraldehyde in 0.2 M PBS (pH 7.4), and dehydrated through an ethanol gradient followed by critical point drying (EMITECH K850 critical point dryer). Samples were coated with gold using an Emscope SC500 sputter coater, and imaged using a Hitachi S2600N Scanning Electron Microscope using with a secondary electron detector (Vacuum 15 kV, electron Beam 50). SEM images were pseudo‐colored using MountainsMap SEM Color 7.3.7984.

### Large Animal Studies

All pig studies were approved by the Italian Ministry of Health (No. 976/2017‐PR) and performed at Explora Biotech Srl (Italy). Prior to the experiments, the animals were housed in single cages and were subjected to a one‐week acclimatization period following Directive 2010/63/EU. Prior to surgery, animals received 10 mg/kg ketamine (KetaVet 100, MSD, Rome, Italy), 0.5 mg kg^−1^ diazepam (Hospira, Naples, Italy), 0.02 mg kg^−1^ atropine (ATI, Bologna, Italy). Anesthesia was induced by 1–5 mg kg^−1^ ketamine and 0.5 mg kg^−1^ diazepam. After intubation, 2–3% isoflurane (IsoFlo, Esteve, Rome, Italy) was administrated by mask to maintain anesthesia.

*Large Animal Studies—Pre‐Clinical Feasibility Study of SMI*: Two female Landrace pigs, weighting 25–30 kg were utilized for the study. The implantation procedure is described in detail in the supplementary methods (Figure S6, Supporting Information). Briefly, the SMI was implanted in a submuscular tissue plane deep to the rectus abdominis and internal oblique muscles in the anterior abdominal wall of the pig. The fill line was tunneled laterally through a separate incision. Positioning of the device was confirmed with fluoroscopy. A 1% Hyaluronic Acid hydrogel containing an iodixanol emulsion was injected into the device and visualized with fluoroscopy. Animals were euthanized (IV injection of Tanax, 0,3 mL kg^−1^; MSD Animal Health Srl‐Italy), and a post‐mortem dissection was carried out to confirm positioning of the device.

*Large Animal Studies—Preclinical Efficacy Study of SMI*: The objectives of this study were 1) to characterize the FBR to the SMI in a pig model of diabetes and 2) assess suitability of SMI for use as a drug‐delivery device. Four female Landrace pigs, weighting 25–30 kg were enrolled in the study. To induce diabetes animals received a single dose of STZ 150 mg kg^−1^ in citrate buffer at pH 4.5 and administered intravenously while under general anesthesia. An i.v bolus of 5% glucose was administered 1 h after STZ treatment to avoid hypoglycaemia, and the animals were carefully monitored for 12 h after recovery of anesthesia. Blood glucose was recorded daily over the 21‐day time course using a MultiCare glucometer (Biochemical Systems International, Italia). On day 7 after induction of diabetes, animals underwent general anesthesia and received surgical implantation of bilateral SMI devices in the submuscular space of the anterior wall. Devices were filled with an HA hydrogel combined with iodixanol emulsion contrast agent so that filling could be visualized under ultrasound during the procedure. On day 21, the animals were again placed under general anesthesia and selected to receive an injection of insulin in the subcutaneous space or an infusion in the device. For animals receiving an infusion of insulin through the SMI, the inlet tubing was exposed and 10 mL of 1 U kg^−1^ regular insulin (Humilin, Eli Lilly and Co.) Blood glucose was monitored at 2 h following insulin delivery. Animals were then euthanized by IV administration of Tanax (0.3 mL kg^−1^; MSD Animal Health Srl‐Italy).

*Large Animal Studies—Micro C/T of SMI and Porcine Soft Tissue*: A full sized SMI device was imaged in a Micro C/T 100 scanner at 45 kVp and 200 µA with a 0.1 mm aluminum filter and a voxel size of 102.6 µm. DICOM images of the resulting scan were segmented in 3D slicer and 3D model of the SMI was generated. The targeted submuscular implant site was identified on axial views of a previously obtained non contrast abdominal C/T scan of a euthanized Landrace swine (Explora Biotech Srl) C/T scan using 3D Slicer and a full‐size 3D model of the SMI was superimposed over the C/T images in the submuscular plane. For imaging of the explanted SMI and surrounding tissue, a gradient ethanol concentration fixation was modified.^[^ ^47^ ^]^ After fixation, the core biopsy samples were taken and the metal markers in the device were dissected and removed from the sample. The tissue was dehydrated and stained in 2% w/v iodine solution in absolute ethanol for 5 days, washed in 100% ethanol to remove excess iodine, then imaged in absolute ethanol. Micro C/T images were captured at 90 kVp and 116 µA with a 0.1 mm Copper filter and voxel size of 88 and 34.13 µm. Images and videos were generated using ImageJ software.

*Large Animal Studies—Tissue Processing and Histology*: Following euthanasia, the devices were removed en bloc with surrounding muscle tissue and fixed in 4% paraformaldehyde. Core biopsy samples were taken systematically at five locations across the device using an 8 mm punch biopsy, placed in a 2% agarose mold to maintain structure, then embedded in paraffin wax blocks. Sections of 5–10 µm were cut and stained with Masson's trichrome for fibrous capsule assessment.

*Large Animal Studies—Pull‐Off Testing of Integrated Ropecoil Device and Silicone Tubing*: A Zwick mechanical testing machine (Z050, Zwick/Roell) with a 100 N load cell was used for pull‐off testing.^[^ ^49^ ^]^ Explanted tissue with integrated ropecoil devices were cut into 1.5 × 1 × 0.5 cm samples. 0.5 cm of the ropecoil device was carefully decoupled from the underlying tissue on both ends of the length of the sample. The 0.32 cm diameter silicone fill line was used a control whereby the tissue area in contact with the tubing is equal to the ropecoil (1 × 0.5 cm). The samples were mounted in the tensile tester using pneumatic clamps at 90 PSI run at a shear rate of 20 mm min^−1^ and maximum tangential adhesion force was recorded (*n* = 3/group).

### Statistical Analysis—Statistical Analysis of Histology

GraphPad Prism (8.1.0) was used for statistical analysis. Normality of distribution was assessed by the Shapiro‐Wilk test. Subsequent parametric and/or non‐parametric tests were performed. For parametric data, an unpaired t‐test was performed for comparing between two groups and a one‐way or two‐way analysis of variance (ANOVA) with post‐hoc Tukey's multiple comparison for comparing between groups. For non‐parametric data, a Mann‐Whitney U was performed for comparing between two groups and a Kruskal–Wallis test for comparing more than two groups. Statistical significance was accepted when P<0.05. A minimum of two blinded counters were used for analysis.

Full details of each test performed, per figure, is given in Table S1, Supporting Information.

*Statistical Analysis—Statistical Analysis of LRC Coating*: Statistical analysis of the coating properties were generally found to display trends without significance. Where appropriate, the p valve is added to the figures, but is omitted where no significance is found. *N* numbers are included in the figure legends.

ANOVA Tables generated in Microsoft Excel are included in Tables S2 and S3, Supporting Information.

## Conflict of Interest

A patent has been filed and published as WO/2018/219590, which relates to creating medical device coatings and therapeutic delivery devices using the liquid rope‐coil and spray deposition techniques.

## Supporting information

Supporting Information

Supplemental Video 1

Supplemental Video 2

## Data Availability

The data that support the findings of this study are available from the corresponding author upon reasonable request.
