# Supplementary material for: Additive Manufacturing of Multi‐Scale Porous Soft Tissue Implants That Encourage Vascularization and Tissue Ingrowth
Source: Adv Healthc Mater. 2021 Jun 24;10(14):2100229. doi: 10.1002/adhm.202100229 (PMC11469332; doi:10.1002/adhm.202100229)
Supplement: Supplementary file 1 — Supporting Information [file ADHM-10-2100229-s003.pdf]

© 2021 Wiley-VCH GmbH

ADVANCED HEALTHCARE MATERIALS

Supporting Information

for *Adv. Healthcare Mater.*, DOI: 10.1002/adhm.202100229

Additive Manufacturing of Multi-scale Porous Soft Tissue Implants that Encourage Vascularization and Tissue Ingrowth

Fergal B. Coulter, Ruth E. Levey, Scott T. Robinson, Eimear B. Dolan, Stefano Deotti, Michael Monaghan, Peter Dockery, Brian S. Coulter, Liam P. Burke, Aoife J. Lowery, Rachel Beatty, Ryan Paetzold, James J. Prendergast, Gabriella Bellavia, Stefania Straino, Francesca Cianfarani, Monica Salamone, Carmelo M. Bruno, Kevin M. Moerman, Giulio Ghersi, Garry P. Duffy, Eoin D. O'Cearbhaill**

Additive Manufacturing of Multi-scale Porous Soft Tissue Implants that Encourage Vascularization and Tissue Ingrowth

Fergal B. Coulter[1†], Ruth E. Levey[2†], Scott T. Robinson[2,3†], Eimear B. Dolan[2,4], Stefano Deotti[1], Michael Monaghan[5], Peter Dockery[2], Brian S. Coulter, Liam P. Burke[6], Aoife J. Lowery[7], Rachel Beatty[2], Ryan Paetzold[1], James J. Prendergast[2], Gabriella Bellavia8, Stefania Straino[8], Francesca Cianfarani[8], Monica Salamone[9], Carmelo M. Bruno[9], Kevin M. Moerman[5,11], Giulio Ghersi[9,10], Garry P. Duffy[2,3,12,*], Eoin D. O'Cearbhaill[1,13,*]

List of Supplementary Materials

Supplementary Materials and Methods:

S1.0 3D printing hardware design

S2.0 Rope-coil parameter analysis

S3.0 Printer parameters for Spray Deposition

S4.0 Quantitative analysis of blood vessels

S5.0 Implantation of drug eluting pouch

Supplemental Figures

Figure S1: 3D Printer Design and Optimisation of Liquid Rope Coiling

Figure S2: Analysis of Curvature, 2D Voids and LCR Coating Heights

Figure S3: Mechanical Integrity of Rope Coil layers

Figure S4: Micropore analysis

Figure S5: Explants seen by transillumination and Micro C/T

Figure S6: Images from surgical procedure

Supplemental Tables

Table S1 : Statistical Analysis methods for Histological analysis.

Table S2: ANOVA for mean loop widths and amplitudes at different deposition heights (varying head speed and material flow rate)

Table S3: Analysis of Curvature, 2D Voids, LCR Coating Heights and Micropore distribution Mean Curvature Analysis

Supplemental Videos

Movie S1: Process Overview & Demonstrator fabrication

Movie S2: Micro/CT of explanted devices

Supporting Information

S1.0 3D printing hardware design

A three axis computer numeric controlled (CNC) gantry (Stepcraft QSeries 420) was customised with a fourth (rotational) axis, heated print platen and a printhead that incorporated a volumetric paste extruder (Viscotec ECOpen EC300), an atomising spray valve (Techcon TS5540 MS) and a measurement laser (Keyence LK-G5000). The various pneumatic and electronic control systems were designed and integrated into a single controller, such that each device was addressable and controlled during the course of a print. The entire assembly was placed inside an enclosure with filtered extraction to remove solvent vapours.

The print head is positioned with precision ball-screws and can repeatably place the toolhead within 0.01 mm in X,Y and Z direction. The A axis can position to within 0.1 minute of a degree. The limiting factor in this case (as with most 3D printers) is the nozzle aperture (and therefore filament diameter) and material flow rate. In this paper we use a 200µm nozzle with a minimum dispensing 'dot size' of 1µL, which limits any feature size that can be printed to at least that volume and diameter.

The quoted scan accuracy of the Keyence is 800nm though this accuracy is dependent on the specularity and absorbance of the substrate being scanned. Empirically we see this accuracy as being

to within 2µm in the Z direction. As discussed above, the scanning system requires the substrate surface to be sampled and computed with interpolated, then lofted curves, which can introduce an inaccuracy

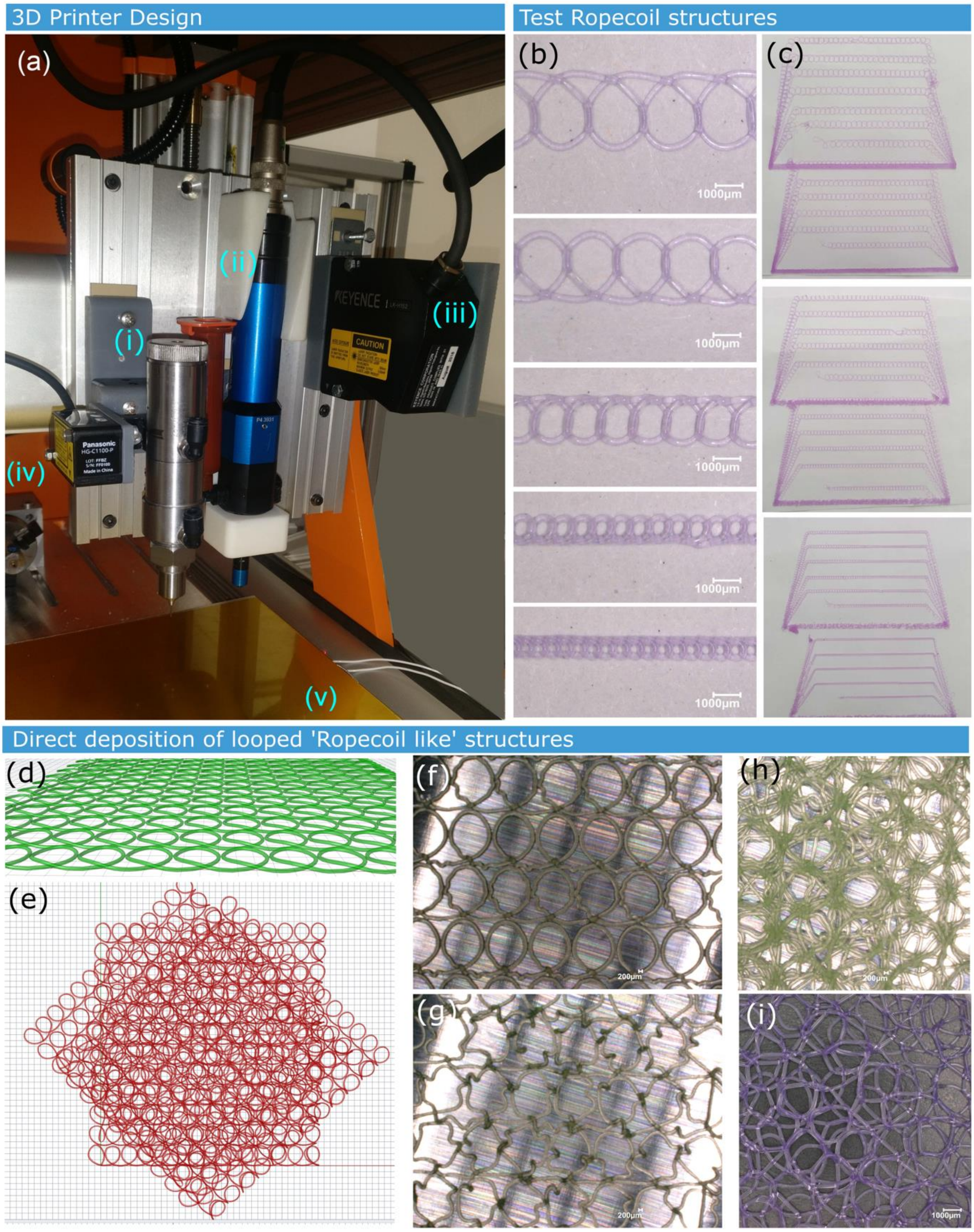

Figure S1: 3D Printer Design and Optimisation of Liquid Rope Coiling (A) 3D Printer Design. (B) Close up of Rope Coil structures used to calculate loop widths and heights. (C) Zoomed out version of various rope coil test structures (D) Toolpath used when printing is attempted using an explicit looping toolpath – Oblique view of a single layer (E) Three Layer full toolpaths (F) Example of a single layer when printed using explicit toolpath – nozzle at 200um above substrate (G) Example of how the same toolpath prints when print head is 600um above substrate (H) Sample output for three-layer explicit toolpath looped 'rope-coil like' structure (I) Example of three-layer Liquid Rope Coil coating.

S2.0 Rope-coil parameter analysis

Parameters for rope-coil layers

Printing parameters used in implants were dependant on the material required:

MED4820 : Q = 140 µl/min, V = 620mm/min, H = 9mm.

MED4840 : Q = 160 µl/min, V = 640mm/min, H = 9mm.

Following deposition, the silicone is cured at 120˚C for 12 minutes.

To compare a Liquid Rope Coil Coating to that of a similar looped geometry but printed with a traditional explicitly specified toolpath, we first create a toolpath with a looped form as per Fig S1 (C). Note the slight lift of the print nozzle at the intersection points for each loop. The toolpath is duplicated and rotated by ±45° and moved to 400 µm and 600 µm in the Z direction, as shown in Fig S1 (D). The first layer (printed with nozzle at 200 µm height) is similar to a standard single layer when deposited using the liquid rope coil effect – Fig S1 (F). Subsequent layers do not print so smoothly, as there is little to no support structure underneath. To make this absolutely obvious, we show what happens when the print-head is positioned 600 µm from the substrate in Fig S1 (G). A three-layer structure, when printed with explicit toolpath is shown Fig S1 (H). Even though there is an underlying layer structure at 0 µm and 200 µm, the porosity is such that there is little to support stacked layers. In the end, we see wavy, uneven lines stacking on top of each other. By way of comparison, a three-layer structure printed by harnessing the liquid rope coil effect is shown in Fig S1 (I)

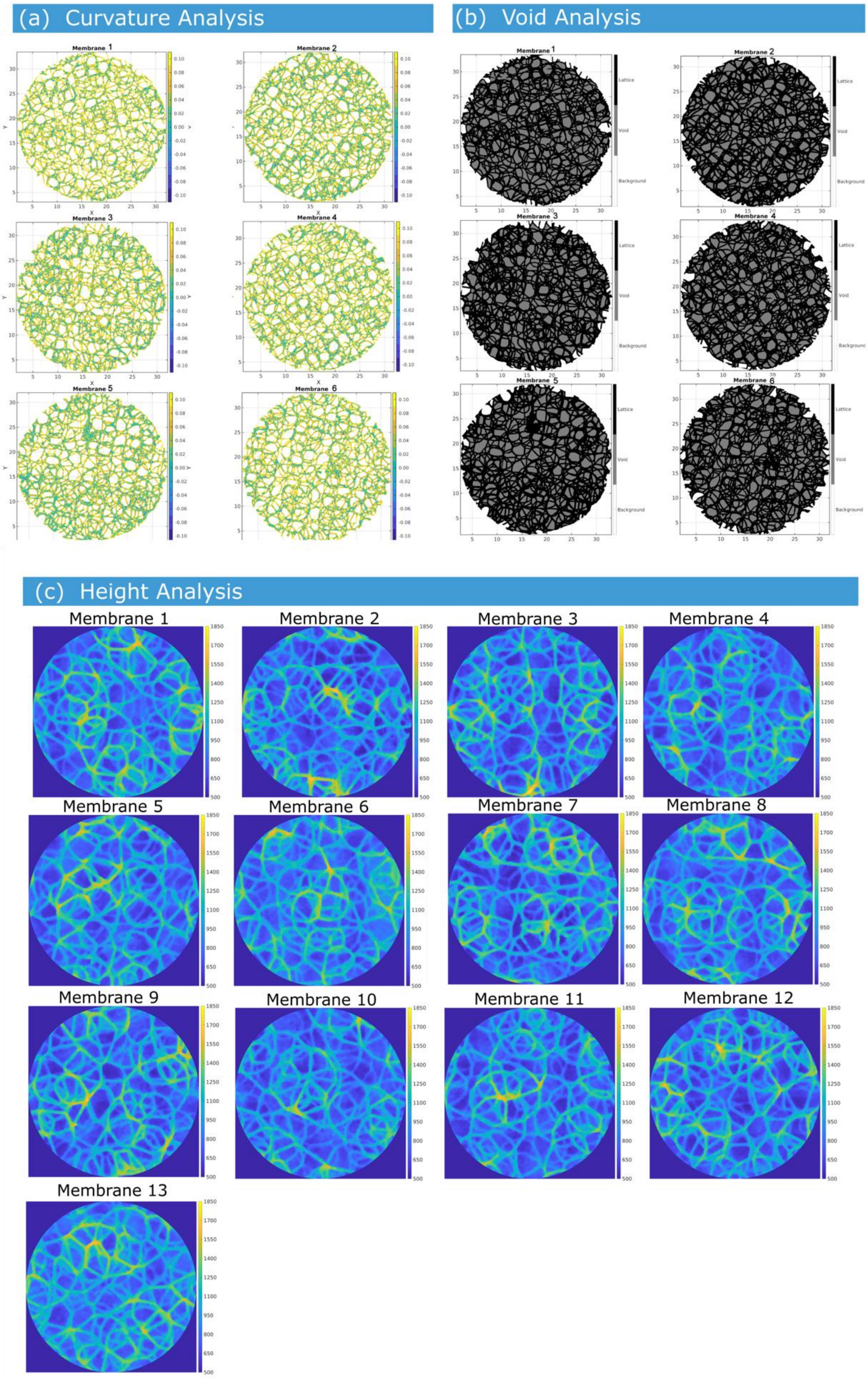

Figure S2: Analysis of Curvature, 2D Voids, LCR Coating Heights and Micropore distribution. (A) Representative Images of LRC Coating Curvature Analysis. (B) Representative Images of LRC Coating 2D Void Analysis. (C) Representative Images of LRC Coating height analysis.

S2.1 Curvature, Height and Void Analysis

Confirmation of repeatability across multiple LRC coatings was ascertained as described in the main paper. With curvature analysis - we see from box-plots in Fig. 1N that there is a definite trend. An ANOVA was performed on the data, but this did not show statistical significance. Instead we saw two distinct populations. The first was Membranes 1-4 ($p=0.6386$), and second 5 & 6 ($p=0.5838$). This distinction between populations can be explained by a manufacturing defect which was not noticed until after tests were performed, which could be eliminated in the future by improved in-process monitoring. In Fig S2 (A), we see a hyper-dense lump of material (green) in membrane 5 & 6. This problem occurs when a small air bubble creates a discontinuity in the flow of material. This results in material sticking to, and bunching up on the nozzle tip for a moment during printing. This lump eventually falls off, and rope-coiling continues as normal. The transparency of the silicone makes visual inspection of these defects challenging, and as such a more reliable in-process quality control method when future scale-up in a more controlled manufacturing environment is considered.

S2.2 Mechanical integrity of Rope Coil layers

To confirm the mechanical integrity of deposited rope coil layers in particular their ability to bond to an underlying substrate, a set of multi-layer test tokens were fabricated and tested using a mechanical tensile tester (Zwick/Roell Z5050). For each test piece two aluminium squares were coated with a silicone membrane (NuSil MED 4840) and cured. The two pieces were placed side by side in the 3D printer and a series of three stacked rope-coil layers (NuSil MED 4820) were extruded upon the membrane. The first layer was either oriented in the direction of or perpendicular to the direction of force. The subsequent two layers were extruded at 45° and then 135° to the force. Such a layer and material configuration corresponds to the fabrication of the implanted device, described later in *Fig. 7*. Samples were subsequently stretched in tension at 20 mm/min (as described in 4.11.5) and in all cases achieved a strain at failure in excess of 100% (*Fig. S3).* The average maximum force was 3.601±0.59 N when LRC orientation was in the direction of the force and 6.936±1.27 N

when LRC was perpendicular to the direction of force (**p=0.0021, n=5/group, Fig. S3(A-D)). Importantly, the failure mode in all cases was the sequential breaking of rope coil fibres, while no debonding from the substrate was observed. This is indicative, as would be expected, that a covalent bond is achieved between the rope coiled layers and the underlying substrate of the same material. The average force at first failure (break of first rope coil fibre) was defined as >2% drop in force during testing. The average force at first failure was 3.05±0.30 N when LRC orientation was in the direction of the force and 5.118±1.32 N when LRC was perpendicular to the direction of force (**p=0.0079, n=5/group, Fig. S3(E)). Cyclical testing was performed (n=6) as described in ASTM 412. Samples were cyclically tested to 100% strain and two feed-rates were used (20 min/min for 20 cycles and 500 mm/min for 50 cycles). Representative curves are shown in Fig. S3(F), hysteresis was observed with no difference between feed-rates. Samples were examined after cyclical testing and there was no evidence of rope coil delamination from the underlying substrate.

Performance of the ropecoil layers under oscillatory shear was examined by selecting the first three samples from the collection illustrated in Fig S2(c). These samples were tested using a Rheometer (Anton Paar MCR502e) with a 2% Conical plate geometry with roughened surface. The tests were performed at a constant distance of 0.8mm - therefore displacement controlled. This distance was chosen as corresponding approximately to the third quartile of measured heights across the samples.

An amplitude sweep was first performed at a constant frequency. Sweeps were performed up to 10% shear strain. The Storage Modulus (G’) and Loss Modulus (G’’) are plotted in Fig S3(G). Subsequent tests were performed, where the sample was firstly subjected to 1% shear strain at 1Hz for 6 hours, followed by 10% shear strain at 10Hz for 1 hour. These plots are shown in Fig S3(H and I). We can see from these graphs that both G’ and G’’ remain constant for each sample over the course of the experiment, thus demonstrating that the coating is not delaminating and remaining coherent when subjected to higher local shear than might be expected for a sub muscular implant.

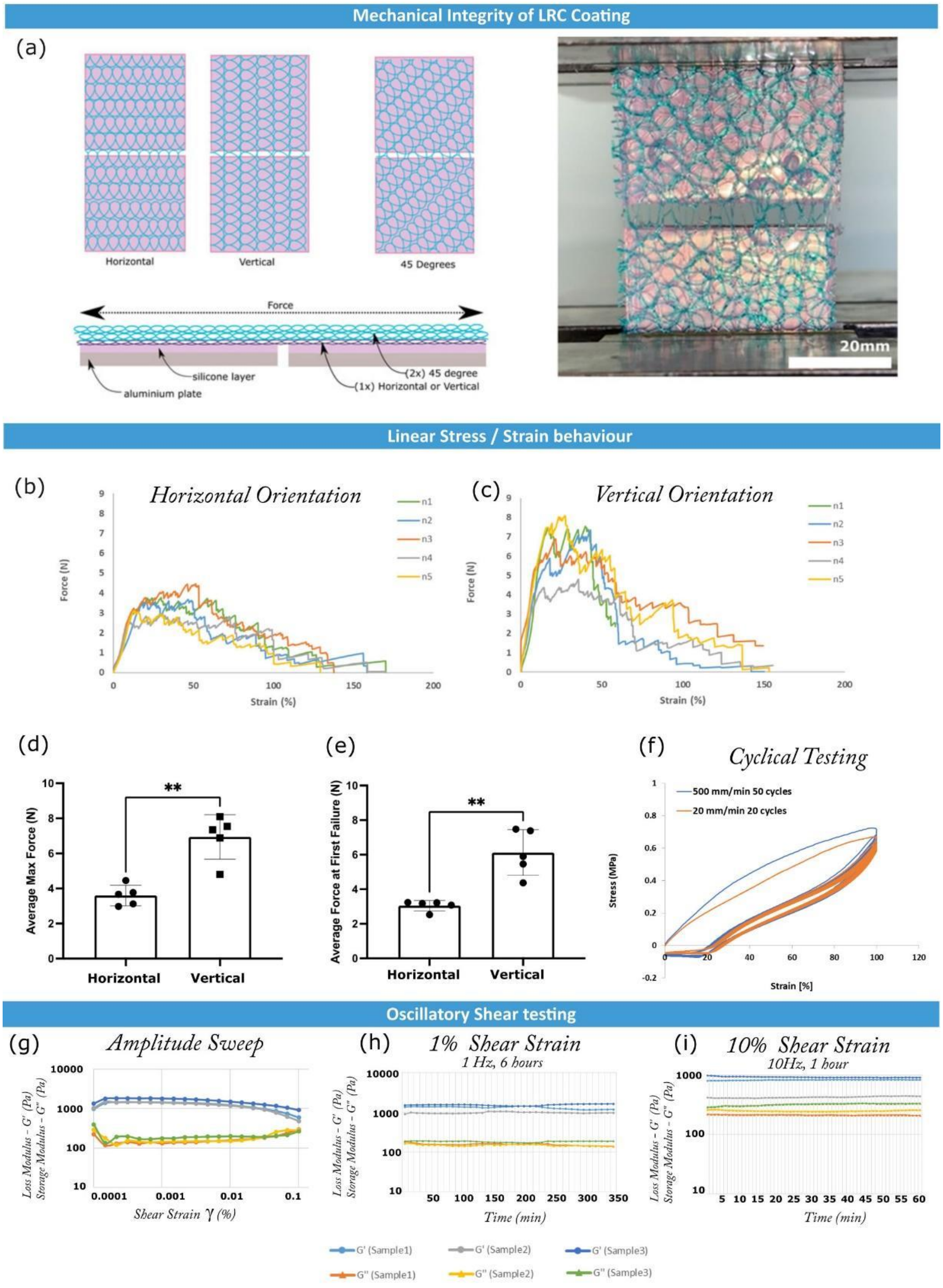

Figure S3: Mechanical Testing of Coating Integrity (A) Schematic of test specimen for Horizontal & Vertical LRC coating test. (B) Stress strain behaviour of Horizontal LRC Coating. Spikes in data are seen as each loop breaks. (C) Stress strain behaviour of Vertical LRC Coating (D) Average Maximum Force, n=6, p<0.01 (E) Average force at first filament break. n=6, p<0.01 (F) Cyclic testing of Substrate with bonded LRC coating, n=3 (G) Amplitude Sweep showing Loss and Storage Modulus against Shear Strain, n=3. (H) 1% Shear Strain at 1Hz plotted over time (I) 10% Shear Strain at 10Hz plotted over time, n=3.

S3.0 Printer parameters for Spray Deposition

When spraying this emulsion, there are numerous factors that define the final nucleated crystal size including spray nozzle aperture, substrate temperature, material flow rate as well as the overall quantity of surfactant used in the emulsion.

The following settings resulted in a single deposited layer with a 5 µm thickness (post-solvent evaporation)

Material pressure:	0.1 bar	Feed rate:	800 mm/min
Atomizing pressure:	0.15 bar	Nozzle aperture:	0.33 mm (23 G)
Substrate temperature:	75˚C	Height from substrate:	85 mm

Provided the silicone substrate surface to be coated is either still in an un-crosslinked state, or else recently cured (where the surface energy has not yet dissipated), then the sprayed coating bonds fully to the underlying layers. *Fig. 2(K)* shows the surface topology when the silicone/salt solution is sprayed on to a warm substrate, then subsequently cured. The nucleated salt crystals are visibly protruding through the silicone surface. *Fig. 2 (I)* shows a close-up of a single salt crystal. The inclusion of the PEG 6000 in the salt water solution has an interesting effect on how the salt crystals nucleate. Rather than the standard ortho-rhombic NaCl crystal a much more rounded, and random crystal emerges. By way of contrast, Fig S4 (B) shows the more classic NaCl crystal when PEG 6000 is not added.

After washing the salt crystals away in an ultrasonic bath, we see a highly porous and complex topology in *Fig. 2 (L)*, where the features are predominantly in the range of 2–8 µm.. Looking closer at the pores, we can see an example in *Fig. 2 (J)*, where the pore created is in the 4–5 µm range. We can also see a complex (random) surface inside the pores.

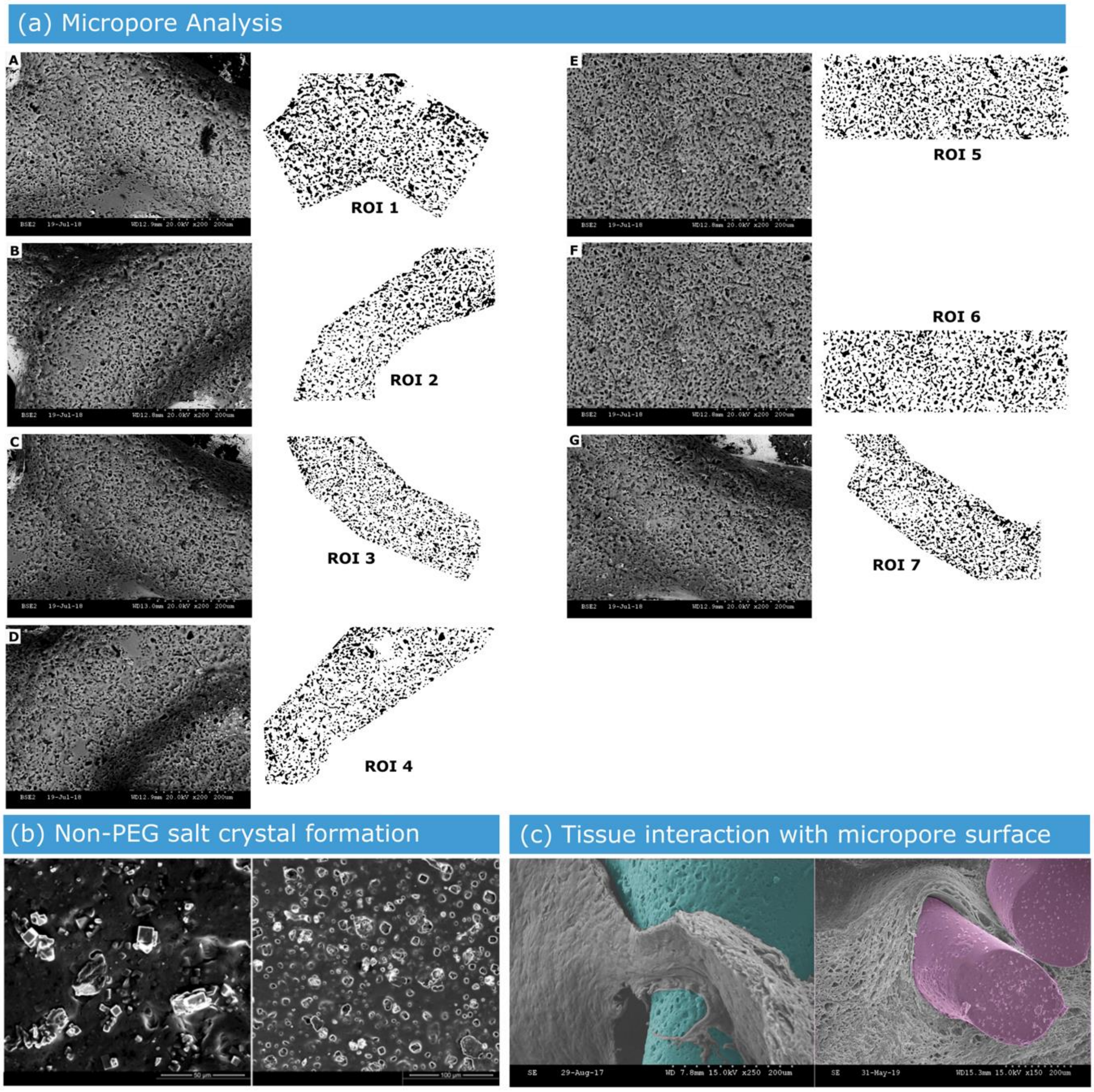

Figure S4 (A) SEM Images of rope-coil layer that has been over sprayed to create a microporous surface texture. The corresponding Region of Interest is shown beside each micrograph. (B) Example of NaCL crystal structures if PEG is not added to ink formulation. (C) Example of tissue integration on a rope coil structure – blue is silicone on top side, pink is silicone on underside, where microporous spray does not deposit.

S4.0 Quantitative analysis of blood vessels

A systematic random sampling strategy was used. From each tissue section, ten non-overlapping images were taken of the tissue surrounding the device at 8× magnification. This tissue included both the immediate fibrous capsule and the tissue extending to the panniculus carnosus

muscle. Using Image J Fiji version 2.0.0, a simple point counting method was used to assess the volume fractions (*Vv*) of the blood vessel. The numbers of points which coincided with blood vessels were counted. The volume fractions of blood vessels were then calculated by expressing the proportion of points hitting blood vessels as a fraction of the total number of points observed in the tissue, from which the final *Vv* was calculated.

Number per unit area, Length Density and Radial Diffusion Distance were all calculated using an unbiased counting grid. This counting frame consists of red forbidden lines and green acceptance lines. Any blood vessel that is cut by a forbidden line is not counted. Blood vessels that appear outside the counting frame or are cut by the acceptance lines without also cutting the forbidden line are counted. The application of this counting rule generates an unbiased estimate of the number of blood vessels per unit area. Number per unit area describes the number of blood vessels per unit area. It is estimated by dividing the number of blood vessels counted by the size of the area viewed. The length density of a blood vessel is the length of blood vessels present per unit volume. It is estimated by multiplying the number per unit area by 2. The radial diffusion distance provides a simple estimation of the cylindrical perfusion space surrounding a blood vessel.

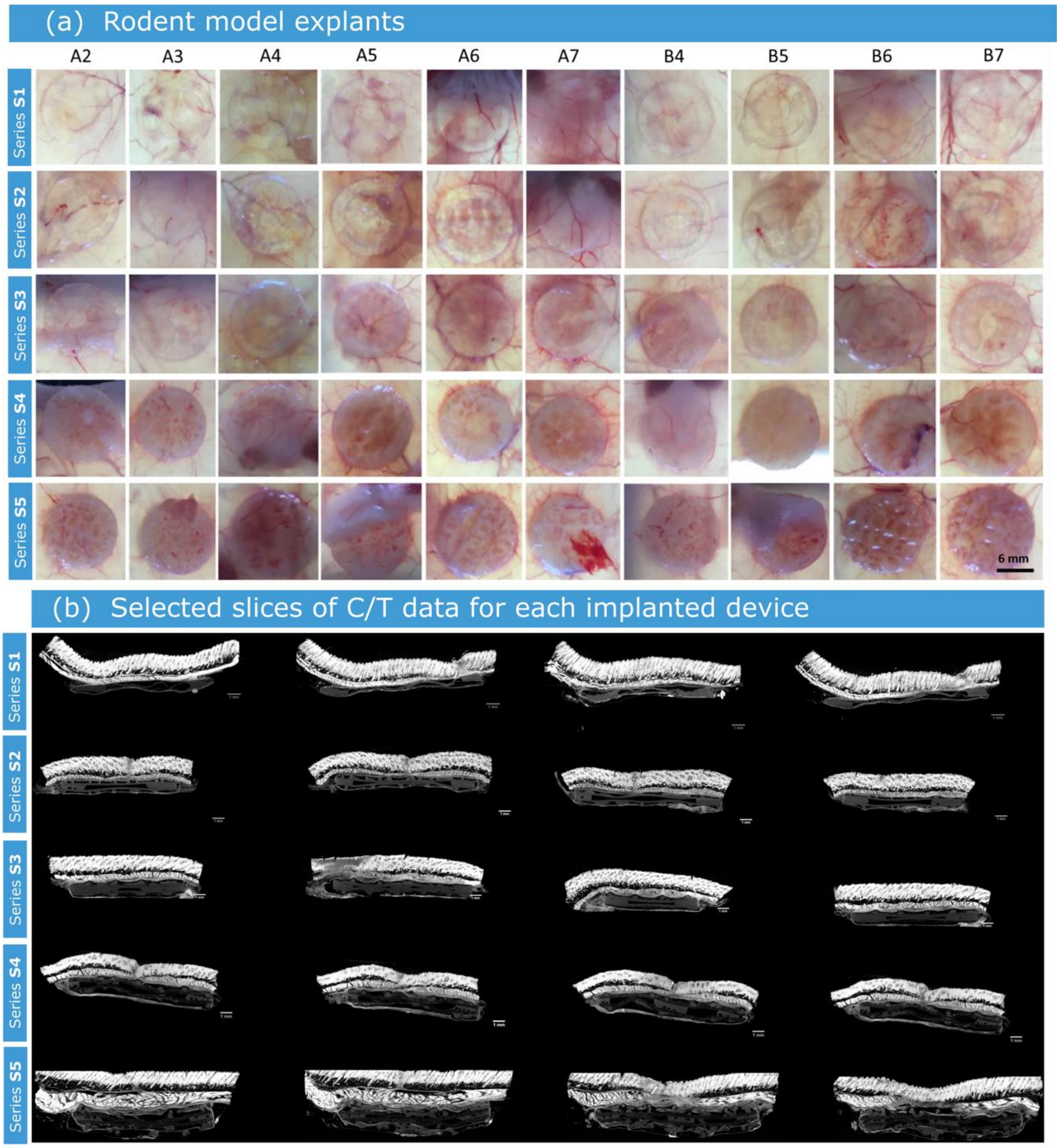

Figure S5 (A) Transilluminated explants from all tested rodents. (B) Selected slices of C/T imagery from each type of implant.

S4.0 Fabrication of drug eluting pouch

The drug eluting pouch was constructed of two porous membranes with a rope-coil layer of NuSil MED4840 (Amplitude of 4.5mm) extruded onto the surface. This is followed by two rope-coil layers at 45° and 135° followed by over-spraying two salt/silicone solution layers. A layer containing an internal support structure was incorporated by extrusion of MED4840 in a pattern that

strengthened the device, prevented ballooning, and directed distal filling of the device cavity with an internal catheter The drug eluting pouch also included a catheter attachment with one-way valve for unidirectional filling and fixation rings reinforced with surgical stainless steel to allow ease of handling and suture fixation of the device without risking damage to the body of the implant.

S5.0 Implantation of drug eluting pouch

A submuscular implant site was used for preclinical efficacy testing of the drug eluting pouch (*Fig. S6*). To implant the device, a vertical incision was made just off midline and the dissection was carried down to the transversus abdominis fascia. A second vertical incision was made 15 cm lateral to the initial decision, and the dissection carried down to the transversus abdominis muscle. The space between the two incisions was bluntly dissected, and a rolled device was brought onto the surgical field with preloaded filling catheter placed. A PDMS loop was tied through the distal suture ring, and the device was passed through from the lateral to the medial incision, and then unfolded and positioned. Sutures were used to secure the device to underlying tissue through 4 suture rings. The fill line was then tunnelled through a 3rd, smaller lateral incision. Filling of the device was achieved through incremental filling of the inner fill line. Two mL of HA emulsion was injected (with either Iodixanol or PFDs). The fill line was then cut, plugged, and buried in the subcutaneous space.

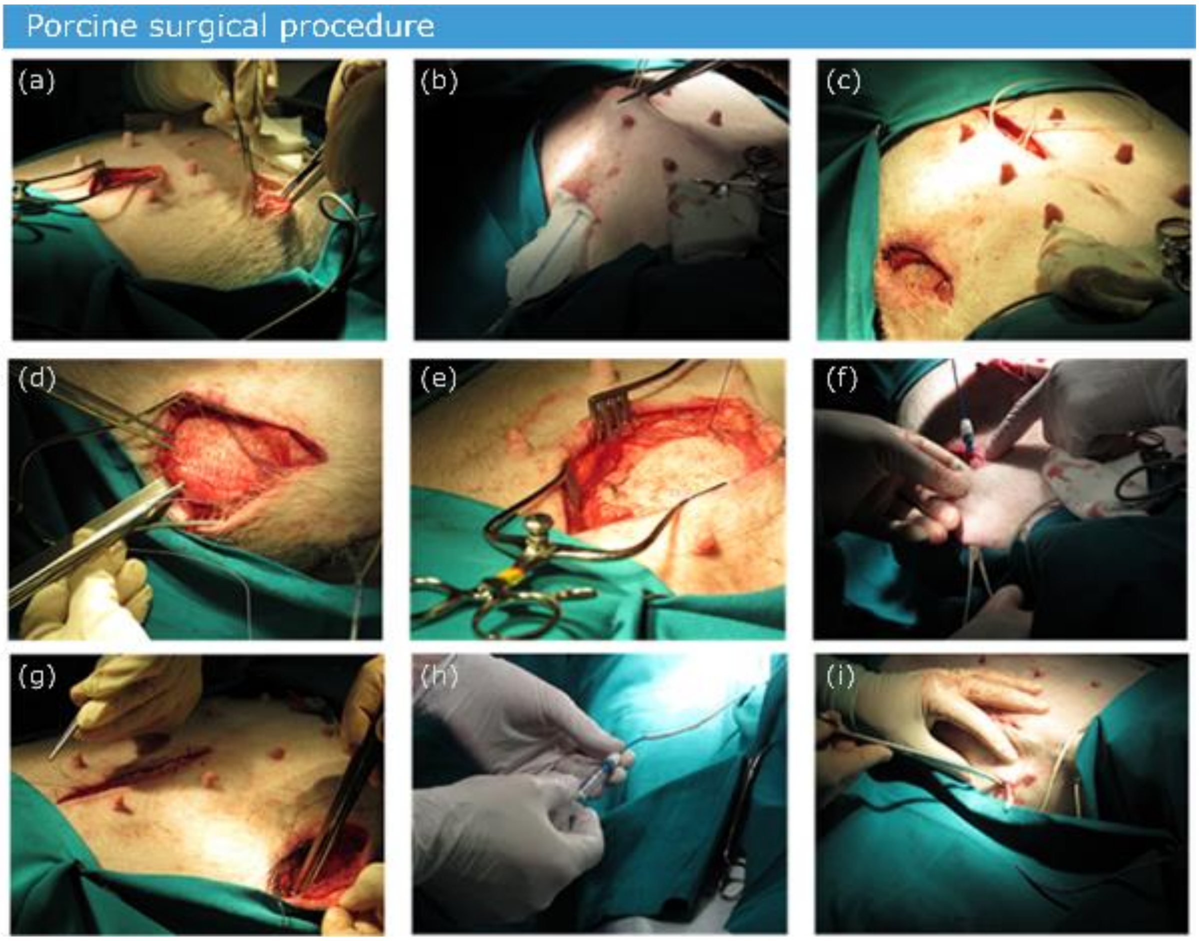

Figure S6: Images from surgical procedure **(a)** Incision and counter incision **(b)** device being pulled through incisions **(c)** positioning of device under rectus muscle **(d)** securing device to underlying tissue via suture rings **(e)** device sutured in place **(f)** tunnelling of infusion line in subcutaneous space **(g)** closure of incisions **(h)** filling of device **(i)** burying tubing in subcutaneous pocket**.**

Table S1 : Statistical Analysis methods for Histological analysis.

**Figure 6**		**n number**	**software**	**Normality**		
D	Fibrous Capsule Thickness	5	GraphPad Prism (8.1.0)	Shapiro Wilk Test	One way ANOVA	Tukeys Multiple Comparisons test
E	Area of fibrous capsule	5		Shapiro Wilk Test	One way ANOVA	Kruskal-Wallis test

F	Volume fraction of CD68 cells	5		Shapiro Wilk Test	One way ANOVA	Tukeys Multiple Comparisons test
G	Volume fraction of M1 to M2-like macrophages	5		Shapiro Wilk Test	Two-way ANOVA	Multiple Comparisons test
**Figure 7**						
C	Volume Fraction of Blood Vessels	5	GraphPad Prism (8.1.0)	Shapiro Wilk Test	One way ANOVA	Tukeys Multiple Comparisons test
D	Radial Diffusion Distance	5				
E	Length Density of blood vessels	5				
F	Number of vessels per area	5				
G	Ratio of α-SMA to CD31+ vessels.	5		Shapiro Wilk Test	One way ANOVA	Kruskal-Wallis test
**Figure 8**						
L	Subcutaneous insulin injection vs infusion through the implant	2	GraphPad Prism (8.1.0)	n too small		
M	Pull-off testing of control smooth silicone and textured implant after 2 weeks of submuscular implantation	3		n too small	two tailed paired t-test	

Table S2: ANOVA for mean loop widths and amplitudes at different deposition heights (varying head speed and material flow rate)

Mean loop widths (h=3mm)

Flow Rate (μl/min)	Head Speed (mm/min) 600	800	1000	1200	Mean
**140**	7.63	7.42	7.59	7.55	7.55
**180**	7.39	7.68	7.64	8.00	7.68
**220**	7.55	7.92	7.78	7.86	7.78
**260**	7.42	7.68	8.29	8.25	7.91
**Overall**	7.50	7.67	7.83	7.91	

ANOVA

Source of Variation	*SS*	*df*	*MS*	*F*	*P-value*
Flow Rate	1.120734	3	0.373578	2.793601	0.050258
Head Speed	1.598339	3	0.53278	3.984105	0.012972
Interaction	1.768998	9	0.196555	1.469833	0.186401
Error	6.418862	48	0.133726		
Total	10.90693	63			

Mean loop amplitudes (h=3mm)

Flow Rate (μl/min)	Head Speed (mm/min) 600	800	1000	1200	Mean
**140**	10.25	11.68	11.84	11.26	11.26
**180**	9.14	10.21	11.14	11.82	10.58
**220**	10.05	10.32	11.29	11.47	10.78
**260**	10.25	10.53	10.91	11.18	10.72
**Overall**	9.92	10.69	11.29	11.43	

ANOVA

Source of Variation	*SS*	*df*	*MS*	*F*	*P-value*
Flow Rate	3.130283	3	1.043428	10.95598	4.16E-05
Head Spee	17.09687	3	5.698956	59.83897	3.22E-13
Interaction	5.66865	9	0.62985	6.613418	2.75E-05
Error	3.047622	32	0.095238		
Total	28.94342	47			

Mean loop widths (h=6mm)

Flow Rate (μl/min)	Head Speed (mm/min) 600	800	1000	1200	Mean
**140**	14.68	12.90	11.56	11.63	12.69
**180**	14.18	14.15	12.98	12.22	13.38
**220**	16.37	14.32	12.98	13.50	14.29
**260**	16.94	14.87	14.87	14.45	15.28
**Overall**	15.54	14.06	13.09	12.95	

ANOVA

Source of Variation	*SS*	*df*	*MS*	*F*	*P-value*
Flow Rate	60.65413	3	20.21804	48.79802	1.29E-14
Head Spee	68.50907	3	22.83636	55.11755	1.41E-15
Interaction	10.0846	9	1.120511	2.704451	0.012432
Error	19.88741	48	0.414321		
Total	159.1352	63			

Mean loop amplitudes (h=6mm)

Flow Rate (μl/min)	Head Speed (mm/min) 600	800	1000	1200	Mean
**140**	21.62	21.21	23.53	24.10	22.62
**180**	21.91	22.01	23.58	23.54	22.76
**220**	23.31	23.21	24.02	25.29	23.96
**260**	22.82	23.84	24.97	25.74	24.34
**Overall**	22.42	22.57	24.02	24.67	

ANOVA

Source of Variation	*SS*	*df*	*MS*	*F*	*P-value*
Flow Rate	26.66475	3	8.888251	36.91351	1.67E-10
Head Spee	43.88703	3	14.62901	60.75528	2.62E-13
Interaction	4.814883	9	0.534987	2.221838	0.04686
Error	7.705147	32	0.240786		
Total	83.07181	47			

Mean loop widths (h=9mm)

Flow Rate (μl/min)	Head Speed (mm/min) 600	800	1000	1200	Mean
**140**	17.841	16.28675	15.73325	16.62033	16.62033
**180**	18.17	18.89	17.21	14.37	17.16
**220**	20.43	19.90	18.64	16.98	18.99
**260**	22.10	19.89	19.24	18.40	19.91
**Overall**	19.64	18.74	17.71	16.59	

ANOVA

Source of Variation	*SS*	*df*	*MS*	*F*	*P-value*
Flow Rate	113.7177	3	37.90591	69.74152	1.62E-17
Head Spee	82.87638	3	27.62546	50.82693	6.2E-15
Interaction	32.26165	9	3.584628	6.595207	4.57E-06
Error	26.08896	48	0.54352		
Total	254.9447	63			

Mean loop amplitudes (h=9mm)

Flow Rate (μl/min)	Head Speed (mm/min) 600	800	1000	1200	Mean
**140**	26.63	26.70	27.71	27.01	27.01
**180**	27.52	27.92	28.55	28.63	28.16
**220**	27.67	28.59	28.67	28.86	28.45
**260**	28.48	28.96	28.59	29.54	28.89
**Overall**	27.57	28.04	28.38	28.51	

ANOVA

Source of Variation	*SS*	*df*	*MS*	*F*	*P-value*
Flow Rate	23.14967	3	7.716556	5.207164	0.004833
Head Spee	6.313145	3	2.104382	1.420046	0.255005
Interaction	3.032002	9	0.336889	0.227334	0.988026
Error	47.42116	32	1.481911		
Total	79.91598	47			

Table S3: Analysis of Curvature, 2D Voids, LCR Coating Heights and Micropore distribution

(a) Curvature Analysis

Sample #	Mean	StDev	Median	Quantile 5	Quantile 25	Quantile 50	Quantile 75	Quantile 95
1	0.0385	0.0652	0.0456	-0.0784	0.0039	0.0456	0.0795	0.1311
2	0.0389	0.0654	0.0461	-0.0783	0.0037	0.0461	0.0806	0.1318
3	0.0368	0.0663	0.0435	-0.0817	0.0013	0.0435	0.0784	0.1317
4	0.0384	0.0648	0.0450	-0.0772	0.0043	0.0450	0.0786	0.1310
5	0.0418	0.0662	0.0480	-0.0753	0.0055	0.0480	0.0836	0.1376
6	0.0431	0.0644	0.0494	-0.0704	0.0079	0.0494	0.0832	0.1355

ANOVA (All 6 groups)

Source of Variation	*SS*	*df*	*MS*	*F*	*P-value*	*F crit*
Between Groups	0.000277	5	5.54E-05	**6.857043**	**4.1964E-05**	**2.370977**
Within Groups	0.000476	59	8.07E-06			
Total	0.000753	64				

ANOVA (Groups 1 to 4)

Source of Variation	*SS*	*df*	*MS*	*F*	*P-value*	*F crit*
Between Groups	1.27E-05	3	4.22E-06	**0.569478**	**0.63836196**	**2.838745**
Within Groups	0.000297	40	7.42E-06			
Total	0.000309	43				

ANOVA (Groups 5 &6)

Source of Variation	*SS*	*df*	*MS*	*F*	*P-value*	*F crit*
Between Groups	2.94E-06	1	2.94E-06	**0.310497**	**0.58388063**	**4.38075**
Within Groups	0.00018	19	9.46E-06			
Total	0.000183	20				

(b) 2D Void Analysis

Sample #	Mean	StDev	Median	Quantile 5	Quantile 25	Quantile 50	Quantile 75	Quantile 95
1	0.4995	0.5508	0.2840	0.0698	0.1432	0.2840	0.5952	1.7082
2	0.4361	0.5220	0.2473	0.0687	0.1242	0.2473	0.4872	1.5277
3	0.4904	0.6111	0.2905	0.0698	0.1242	0.2905	0.5857	1.6519
4	0.5006	0.5524	0.2946	0.0722	0.1441	0.2946	0.6357	1.7023
5	0.4762	0.5881	0.2307	0.0698	0.1207	0.2307	0.5588	1.8518
6	0.5099	0.6360	0.2633	0.0730	0.1296	0.2633	0.6354	1.7771

ANOVA

Source of Variation	*SS*	*df*	*MS*	*F*	*P-value*	*F crit*
Between Groups	0.028781	5	0.005756	**1.693881**	**0.166535**	**2.533555**
Within Groups	0.00018	30	0.003398			
Total	0.130728	35				

(c) Coating height analysis

Sample #	Mean	StDev	Median	Quantile 5	Quantile 25	Quantile 50	Quantile 75	Quantile 95
1	969.7518	215.0903	943.4582	672.9562	791.7131	943.4582	1134.7888	1352.5100
2	926.9596	224.0915	887.5219	626.4861	730.9004	887.5219	1096.3506	1331.2829
3	915.3792	241.0058	872.7131	583.9960	708.6693	872.7131	1115.4980	1338.5976
4	952.8900	211.6757	926.1116	665.2351	776.1076	926.1116	1121.7689	1317.4263
5	918.7837	223.8550	875.6175	623.5458	729.6813	875.6175	1094.5219	1326.6932
6	906.7814	208.8657	872.7649	627.8884	728.3506	872.7649	1067.4104	1280.8924
7	913.4252	232.6037	881.8327	604.4821	711.1554	881.8327	1088.0677	1322.7490
8	897.3027	229.5567	863.3625	593.5618	708.2271	863.3625	1058.9681	1315.2789
9	951.5411	227.8259	915.8566	647.6414	752.3108	915.8566	1125.1952	1354.1594
10	912.5854	229.7739	897.6175	600.8845	712.1594	897.6175	1090.4940	1313.0438
11	886.9024	209.9450	848.0080	604.7809	716.5339	848.0080	1051.7928	1255.5777
12	873.9120	197.3928	832.4502	622.7490	711.7131	832.4502	1016.7331	1232.7888
13	917.6615	237.8304	876.8566	614.4900	711.1514	876.8566	1097.7968	1346.3546

ANOVA

Source of Variation	*SS*	*df*	*MS*	*F*	*P-value*	*F crit*
Between Groups	30048.54	9	3338.726	**1.704572**	**0.112721**	**2.073351**
Within Groups	97934.42	50	1958.688			
Total	127983	59				

(d) Micropore distribution analysis - $\sqrt{Area}$ (in µm)

	Median	1st Ventile (D5)	1st Decile (D10)	1st Quartile (D25)	2nd Quartile (D50)	3rd Quartile (D75)	9th Decile (D90)	19th Ventile (D95)	Span (D90-D10)/D50
**ROI-1**	3.8406	1.5811	2.2913	2.5000	3.8406	6.1644	9.1924	11.7686	1.9425
**ROI-2**	4.1833	1.5811	2.3452	2.6458	4.1833	6.9282	10.0772	11.9080	1.9478
**ROI-3**	3.6056	1.6583	2.2361	2.4495	3.6056	5.7228	8.0607	9.8698	1.7274
**ROI-4**	3.8079	1.5811	2.2361	2.5000	3.8079	5.9582	8.6890	10.8408	1.7930
**ROI-5**	4.3875	1.5811	2.3979	2.6458	4.3875	6.3738	9.0802	11.0565	1.6372
**ROI-6**	4.7697	1.7321	2.7386	3.0414	4.7697	7.8422	10.9510	13.5940	1.8486
**ROI-7**	4.0620	1.5811	2.2361	2.5000	4.0620	6.7639	9.2736	11.9478	1.8254
